# Characteristic analysis and identification of novel molecular biomarkers in elderly glioblastoma patients using the 2021 WHO Classification of Central Nervous System Tumors

**DOI:** 10.3389/fnins.2023.1165823

**Published:** 2023-06-09

**Authors:** Yaning Wang, Junlin Li, Yaning Cao, Wenlin Chen, Hao Xing, Xiaopeng Guo, Yixin Shi, Yuekun Wang, Tingyu Liang, Liguo Ye, Delin Liu, Tianrui Yang, Yu Wang, Wenbin Ma

**Affiliations:** ^1^Department of Neurosurgery, Center for Malignant Brain Tumors, National Glioma MDT Alliance, Peking Union Medical College Hospital, Chinese Academy of Medical Sciences and Peking Union Medical College, Beijing, China; ^2^Eight-Year Medical Doctor Program, Chinese Academy of Medical Sciences and Peking Union Medical College, Beijing, China; ^3^China Anti-Cancer Association Specialty Committee of Glioma, Beijing, China

**Keywords:** glioblastoma, geriatric, 2021 WHO Classification of Central Nervous System Tumors, *KRAS*, *PPM1D*

## Abstract

**Introduction:**

Elderly glioblastoma (GBM) patients is characterized by high incidence and poor prognosis. Currently, however, there is still a lack of adequate molecular characterization of elderly GBM patients. The fifth edition of the WHO Classification of Central Nervous System Tumors (WHO5) gives a new classification approach for GBM, and the molecular characteristics of elderly GBM patients need to be investigated under this new framework.

**Methods:**

The clinical and radiological features of patients with different classifications and different ages were compared. Potential prognostic molecular markers in elderly GBM patients under the WHO5 classification were found using Univariate Cox regression and Kaplan–Meier survival analysis.

**Results:**

A total of 226 patients were included in the study. The prognostic differences between younger and elderly GBM patients were more pronounced under the WHO5 classification. Neurological impairment was more common in elderly patients (*p* = 0.001), while intracranial hypertension (*p* = 0.034) and epilepsy (*p* = 0.038) were more common in younger patients. Elderly patients were more likely to have higher Ki-67(*p* = 0.013), and in elderly WHO5 GBM patients, *KMT5B* (*p* = 0.082), *KRAS* (*p* = 0.1) and *PPM1D* (*p* = 0.055) were each associated with overall survival (OS). Among them, *KRAS* and *PPM1D* were found to be prognostic features unique to WHO5 elderly GBM patients.

**Conclusion:**

Our study demonstrates that WHO5 classification can better distinguish the prognosis of elderly and younger GBM. Furthermore, *KRAS* and *PPM1D* may be potential prognostic predictors in WHO5 elderly GBM patients. The specific mechanism of these two genes in elderly GBM remains to be further studied.

## Introduction

Glioma is the most common primary intracranial malignant tumor, and epidemiological statistics show that the incidence of glioma, especially glioblastoma (GBM), increases with age. The incidence of GBM is more than twice as high in people over 65 years of age as in younger people and is highest in people aged 75–84 years (Ostrom et al., 2021). In terms of treatment, the therapeutic effect of elderly GBM patients gradually deteriorates with age (Pretanvil et al., 2017). In consideration of the poor general condition of elderly GBM patients, their tolerance to treatment is far lower than that of younger GBM patients. Therefore, the standard treatment regimen (Stupp et al., 2005) is not fully applicable for elderly patients. At present, there is no consensus on the most appropriate operation and subsequent adjuvant therapy for elderly GBM patients (Wang et al., 2020).

In terms of molecular characteristics, elderly patients differ from younger patients in genome, epigenetics, molecular subtypes, and prognostic-related molecular markers as well (Jones et al., 2012; Ferguson et al., 2016). Moreover, many genes associated with aging also play an important role in elderly GBM patients (López-Otín et al., 2013; Coppola et al., 2014; Chakravarti et al., 2021), although these molecular features have yet to be well studied. With the promotion of precision medicine and the development of sequencing technology, however, molecular markers are playing an increasingly important role in the diagnosis and treatment of GBM (Montégut et al., 2022). Therefore, the exploration of molecular characteristics of elderly GBM patients has become an essential part of the treatment of this subgroup.

Recently, the diagnostic criteria for GBM changed with the publication of the fifth edition of the WHO classification of CNS tumors (WHO CNS 5 classification) in 2021. The new criteria include IDH mutation status, TERT promoter mutation status, EGFR expression, and chromosomal changes, which further illustrate the importance of molecular characteristics in GBM (Louis et al., 2021). Therefore, we analyzed the clinical and molecular characteristics of 98 elderly GBM patients aged ≥60 years in Peking Union Medical College Hospital (PUMCH) based on the new classification. The clinical characteristics of this subgroup of patients were summarized, and we identified several molecular markers that may have predictive value for the prognosis of elderly GBM.

## Methods

### Patient cohort

Participants were screened from the group of glioma patients who underwent surgical resection or biopsy from 2011 to 2022 in Peking Union Medical College Hospital (PUMCH). The inclusion criteria were (1) age ≥18 years, (2) diagnosis of glioma under WHO4 or WHO5 classification, and (3) patients treated by surgical resection or biopsy, (4) Accurate clinical follow-up data can be obtained. The diagnostic criteria are reported below. Exclusion criteria were (1) unconfirmed pathology or without tumor tissue, (2) pregnancy, and (3) nonsurgical resection or biopsy. This study was approved by the Regional Ethics Committee at Peking Union Medical College Hospital (S-424).

### Clinical, radiological, and tumor pathological data collection

A series of clinical information including gender, age, body mass index (BMI), Karnofsky performance status (KPS), clinical symptoms, and extent of surgical resection was collected from the archives of PUMCH. Overall survival (OS) was defined as time from surgery to death or last follow-up date. Based on mutation screening demand, some patient tumor tissues were obtained during surgery and transformed into formalin-fixed paraffin-embedded (FFPE) sections. The radiological profile was collected from preoperative magnetic resonance imaging (MRI) sequences, and imaging characteristics including maximal diameter, tumor location, bleeding, cystic degeneration, calcification, edema, necrosis, and clarity of margin were identified by an experienced neuroradiologist.

### Gene detection

The DNA in all Formalin-fixed paraffin-embedding (FFPE) tumor tissue was extracted using QIAGEN 56404, And the DNA concentration and purity were determined using Qubit 4.0 Fluorometer (Thermo Fisher Scientific) and Nanodrop 2000 spectrophotometer (Thermo Fisher Scientific). Then, we referred to the method proposed by Quail et al. (2008). For DNA fragmentation and PCR amplification. Finally double-ended sequencing was performed by NovaSeq 6000, and the average sequencing depth was required to be greater than 500×. Combining with the results of literature research and WHO 5 classification, a total of 60 genes were selected for detection. Single nucleotide polymorphism (SNP) and indel of genes were defined as sequence changes not found in East Asian healthy population database, Han population database in southern China, and non-tumor population database in GnomAD database, and mutations were required to occur in exon region. The detection method of copy number variation mainly refers to the research of Talevich et al. (2016). CNVkit is used to identify the copy number variation through DNA sequencing results. The SNP, indel, and mutations that cannot be identified as amplification or deletion are defined as mutations. The SNP, indel and deletion or amplification occur at the same time, they are uniformly denoted as deletion or amplification.

### GBM classification

All patients underwent re-classification based on detection results. WHO4 GBM was identified by pathologist under microscopes, and WHO5 GBM was re-classified as IDH wildtype with at least one characteristic of either microvascular proliferation, necrosis, TERT promoter mutation, EGFR amplification, or +7/−10 chromosome copy-number alterations.

### Statistical analysis

In baseline data, continuous variables were expressed by means and standard deviations and compared with Chi-squared tests. Categorical variables were expressed as numbers and percentages and Student’s *t*-test was used to test for differences between groups, and oncoplots were utilized to illustrate biomarker alterations in samples. Univariate Cox regression was used to reveal the impact of individual biomarkers on prognosis, and biomarkers for which regression could not be performed due to insufficient number of alterations are not shown. The Kaplan–Meier method with log-rank test was used to carry out survival analysis implemented in RStudio (PBC & Certified B Corp^®^., United States) and SPSS statistical analysis v26 (IBM Corp., New York, NY, United States). For univariate Cox regression, *p* < 0.1 were considered to indicate statistically significant test results, and for Kaplan–Meier, we used *p* < 0.05.

## Results

### Differences in prognosis among GBM patients using WHO4 and WHO5 classifications

**Figure 1:**
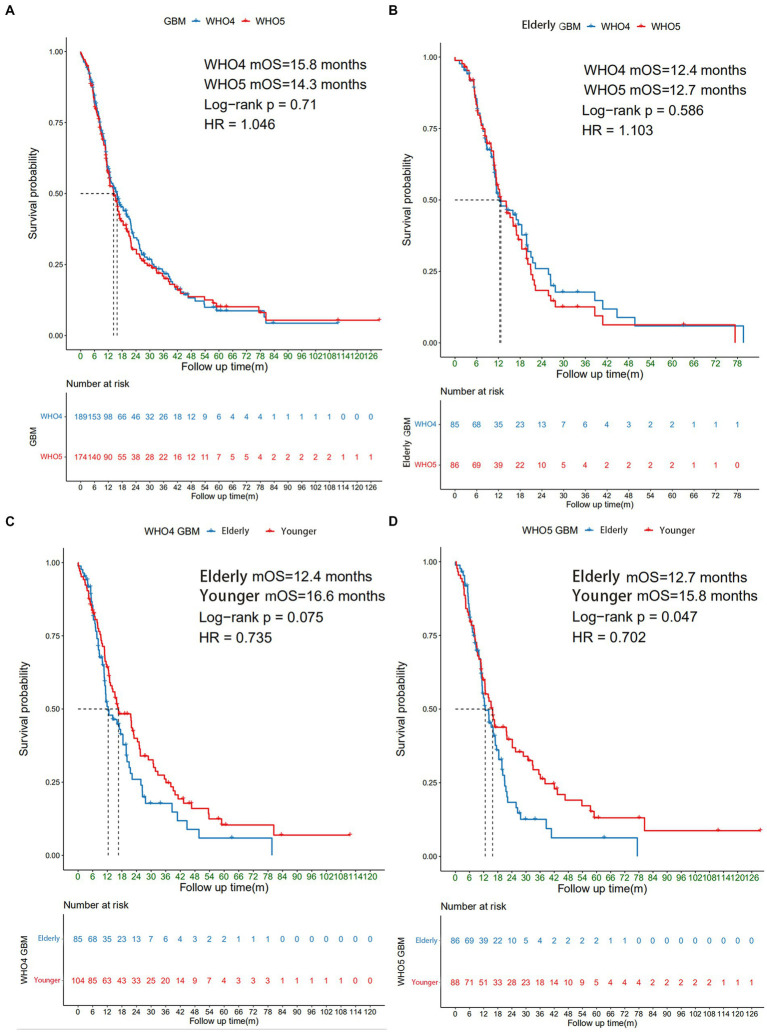
Survival analysis of different subgroups of GBM patients. The Kaplan–Meier curve showing **(A)** all GBM patients under WHO4 and WHO5 classifications; **(B)** elderly GBM patients under WHO4 and WHO5 classifications; **(C)** younger and elderly GBM patients under WHO4 classification; and **(D)** younger and elderly GBM patients under WHO5 classification.

A total of 226 patients with WHO4 or WHO5 GBM who underwent surgery in Peking Union Medical College Hospital (PUMCH) from January, 2011 to December, 2022 were included in this study, 98 of whom were aged ≥60 years. Among all the GBM patients, 137 patients can be diagnosed by both WHO 4 and WHO 5 classification, and 73 of whom were aged ≥60 years. To explore the prognoses and clinical and molecular characteristics of elderly GBM patients under the new classification, Kaplan–Meier survival analysis was used to compare the overall survival (OS) of patients under both WHO4 and WHO5 classifications (Figure 1). For all GBM patients combined (including non-elderly patients), there was no significant difference in median OS (mOS) between WHO4 and WHO5 classifications (15.8 m *VS* 14.3 m, *p* = 0.71, Figure 1A), nor was there one for elderly patients specifically (12.4 m vs. 12.7 m, *p* = 0.586, Figure 1B). We further compared the outcomes of younger and elderly GBM patients under different classifications and found that under the WHO5 classification, the mOS of elderly patients was 12.7 m, while that of younger GBM patients was 15.8 m (*p* = 0.047, Figure 1D). This difference in mOS was even more obvious than that under the WHO4 classification (*p* = 0.075; Figure 1C). Therefore, we concluded that the differences between younger and elderly GBM patients are more significant under the WHO5 classification and that the application of this new CNS classification system may thus be more beneficial for distinguishing the clinical and molecular characteristics of these two subgroups of patients.

### Clinical, radiological, and pathological features of elderly GBM patients

A further subgroup analysis was conducted to explore the clinical, radiological, and pathological characteristics of elderly GBM patients using the WHO5 classification (Table 1). By comparing the clinical features of elderly GBM patients (≥60 years old, *n* = 86) and younger GBM patients (<60 years old, *n* = 88) under the WHO5 classification, we found that the differences between them mostly consisted of clinical symptoms. Neurological impairment was more common in elderly patients (*p* = 0.001), and intracranial hypertension (*p* = 0.034) and epilepsy (*p* = 0.038) were more common in younger patients. We found that the expression level of *Ki-67* was higher in elderly patients than in the younger patients under the WHO5 classification (*p* = 0.013). For radiological features, such as peritumoral edema, tumor necrosis, and intratumoral bleeding, there was no significant difference between the two subgroups. Comparing the elderly GBM patients under different classifications (WHO4, *n* = 85 and WHO5, *n* = 86), there was no significant difference. This is due to the high overlap of patients in these two subgroups. The specific results are shown in Table 1.

**Table 1:** Demographics and clinicopathological characteristics of different subgroups of GBM patents.

	GBM WHO4, ≥60	GBM WHO5, ≥60	GBM* WHO5, <60	*p*_1_ ****	*p*_2_ *****
*n*	**85**	**86**	**88**		
**Gender (%)**				1	1
Female	41 (48.2)	42 (48.8)	43 (48.9)		
Male	44 (51.8)	44 (51.2)	45 (51.1)		
**Mean Age, year (±SD)**	67.61 (7.12)	67.03 (7.06)	45.47 (11.52)	0.596	**<0.001**
**Age(%)**				0.681	**<0.001**
<18	0 (0.0)	0 (0.0)	1 (1.1)		
18–44	0 (0.0)	0 (0.0)	31 (35.2)		
45–64	31 (36.5)	35 (40.7)	56 (63.6)		
≥65	54 (63.5)	51 (59.3)	0 (0.0)		
**Mean BMI** ** **, kg/m**^**2**^**(±SD)**	23.86 (2.97)	23.95 (3.05)	23.64 (3.61)	0.858	0.550
**BMI, kg/m**^**2**^**(%)**				0.691	0.084
<18	0 (0.0)	0 (0.0)	5 (5.7)		
18–24	49 (55.6)	46 (51.2)	44 (50.6)		
≥24	36 (44.4)	40 (48.8)	38 (43.7)		
**Preop. KPS** *** **(±SD)**	81.82 (17.68)	83.55 (15.99)	82.84 (17.32)	0.505	0.781
**Clinical symptoms (%)**					
Intracranial hypertension	32 (37.6)	38 (44.2)	54 (61.4)	0.475	**0.034**
Neurologic impairment	72 (84.7)	70 (81.4)	50 (56.8)	0.709	**0.001**
Epilepsy	12 (14.1)	11 (12.8)	25 (28.4)	0.968	**0.038**
**Post-operation therapy (%)**					
Chemotherapy	43 (87.8)	42 (89.4)	47 (85.5)	1	0.77
Radiotherapy	38 (77.6)	39 (83.0)	40 (72.7)	0.681	0.319
Targeted Therapy	14 (28.6)	11 (23.4)	20 (37.0)	0.731	0.206
**Ki-67 (±SD)**	0.42 (0.24)	0.37 (0.23)	0.28 (0.21)	0.289	**0.013**
**Extent of surgical resection(%)**				0.965	0.357
Gross total resection	50 (58.8)	47 (54.7)	61 (69.3)		
Subtotal resection	4 (4.7)	4 (4.7)	4 (4.5)		
Partial resection	8 (9.4)	11 (12.8)	8 (9.1)		
Biopsy	21 (24.7)	22 (25.6)	14 (15.9)		
**Maximum diameter of the tumor, cm (±SD)**	4.34 (1.59)	4.19 (1.70)	4.93 (7.27)	0.584	0.396
**Number of tumors (%)**				0.908	0.163
Solitary	59 (69.4)	57 (66.3)	67 (76.1)		
Multiple	17 (20.0)	19 (22.1)	10 (11.4)		
**Side of the tumor (%)**				0.928	0.493
Bilateral	4 (4.7)	6 (7.0)	9 (10.2)		
Left hemisphere	46 (54.1)	44 (51.2)	35 (39.8)		
Right hemisphere	26 (30.6)	27 (31.4)	33 (37.5)		
**Imaging (%)**					
Tumor in functional region	44 (51.2)	44 (51.2)	47 (53.4)	0.438	0.885
Intratumoral bleeding	19 (22.1)	19 (22.1)	14 (15.9)	0.914	0.397
Cystic degeneration	31 (36.0)	31 (36.0)	29 (33.0)	1	0.788
Calcification	3 (3.5)	3 (3.5)	6 (6.9)	1	0.505
Edema	73 (84.9)	73 (84.9)	71 (80.7)	0.5	0.594
Intratumoral necrosis	47 (54.7)	47 (54.7)	46 (52.3)	0.765	0.871
Clarity of tumor margin	43 (50.0)	43 (50.0)	46 (52.3)	0.533	0.882
Edema diameter, cm (±SD)	2.23 (1.34)	2.13 (1.40)	2.06 (2.33)	0.665	0.811
Central necrosis diameter of tumor, cm (±SD)	2.97 (1.62)	2.77 (1.63)	2.58 (1.64)	0.469	0.505

* GBM, glioblastoma.

** BMI, body mass index.

*** Preop. KPS, Preoperative Karnofsky Performance Status.

**** *p*_1_: the *p* value obtained by comparison between WHO4 and WHO5 elderly glioblastoma.

***** *p*_2_: the *p* value obtained by comparison between younger and elderly glioblastoma of WHO 5.Those values marked in bold are *p* values ≤ 0.05, indicating significant differences between subgroups.

### Molecular characteristics of elderly GBM patients under the WHO5 classification

**Figure 2:**
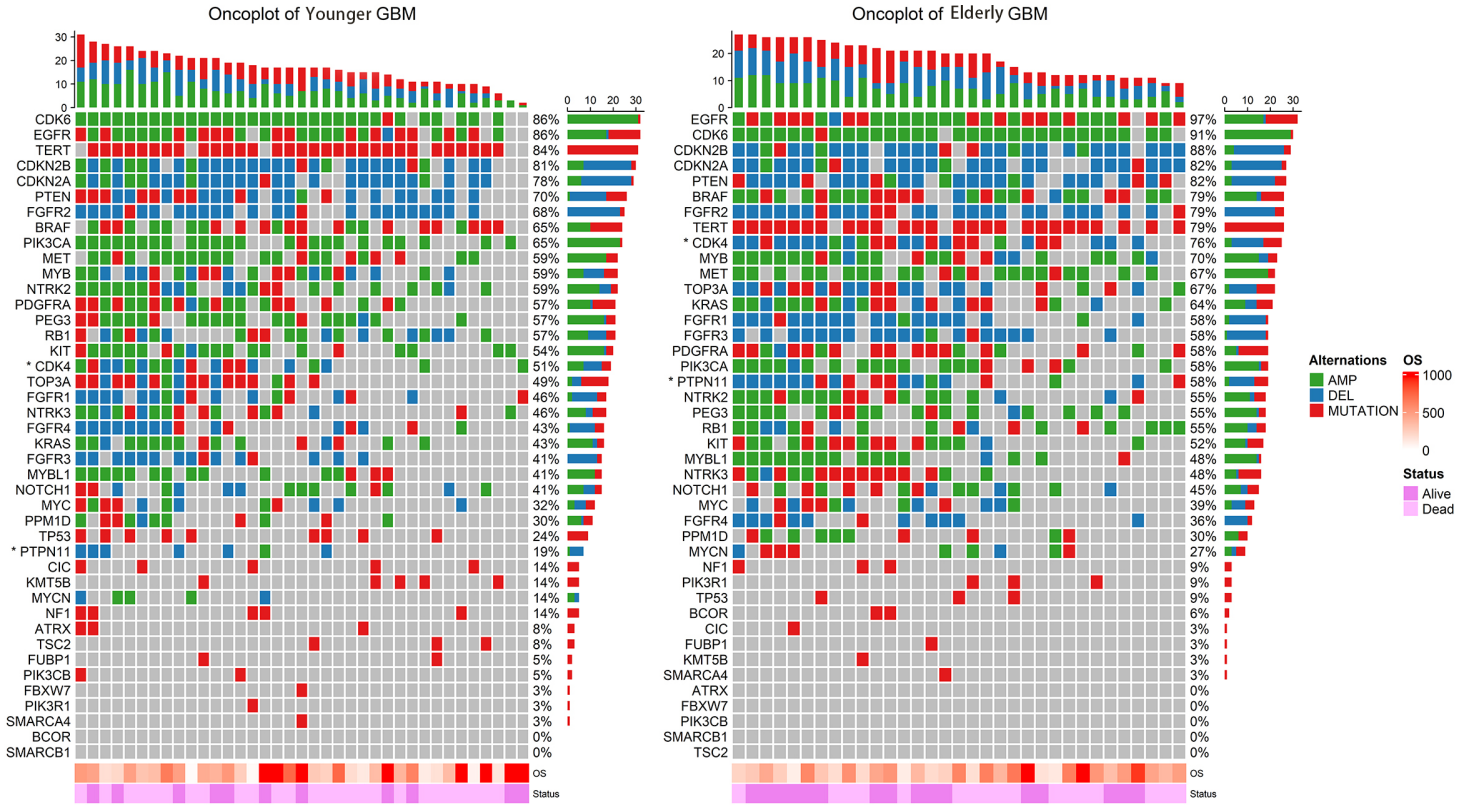
Oncoplot of the alteration of key genes in younger GBM (left) and elderly GBM (right). The rows represent genes with frequency alterations, and the columns represent the samples. OS and survival status are displayed at the bottom. Genes with statistically significant numerical differences between younger GBM and elderly GBM are labelled with an asterisk.

The incidence of GBM increases significantly in the elderly population, and its prognosis deteriorates with age (Ostrom et al., 2021). However, this trend does not seem to be perfectly explained by the differences in the clinical features described above. Therefore, we compared and analyzed the molecular characteristics of 33 elderly patients and 37 younger patients who had had 60 pre-determined molecule biomarkers detected. A detailed molecular landscape of WHO5 GBM patients is shown in Figure 2. Here we can see that there are differences in chromosome and gene alteration, including mutation, deletion, and amplification between younger and elderly patients. The exact number and percentage of specific chromosomal and genetic changes for each subgroup are summarized in Supplementary Table S1.

### Potential prognostic molecular markers in elderly GBM patients under the WHO5 classification

**Figure 3:**
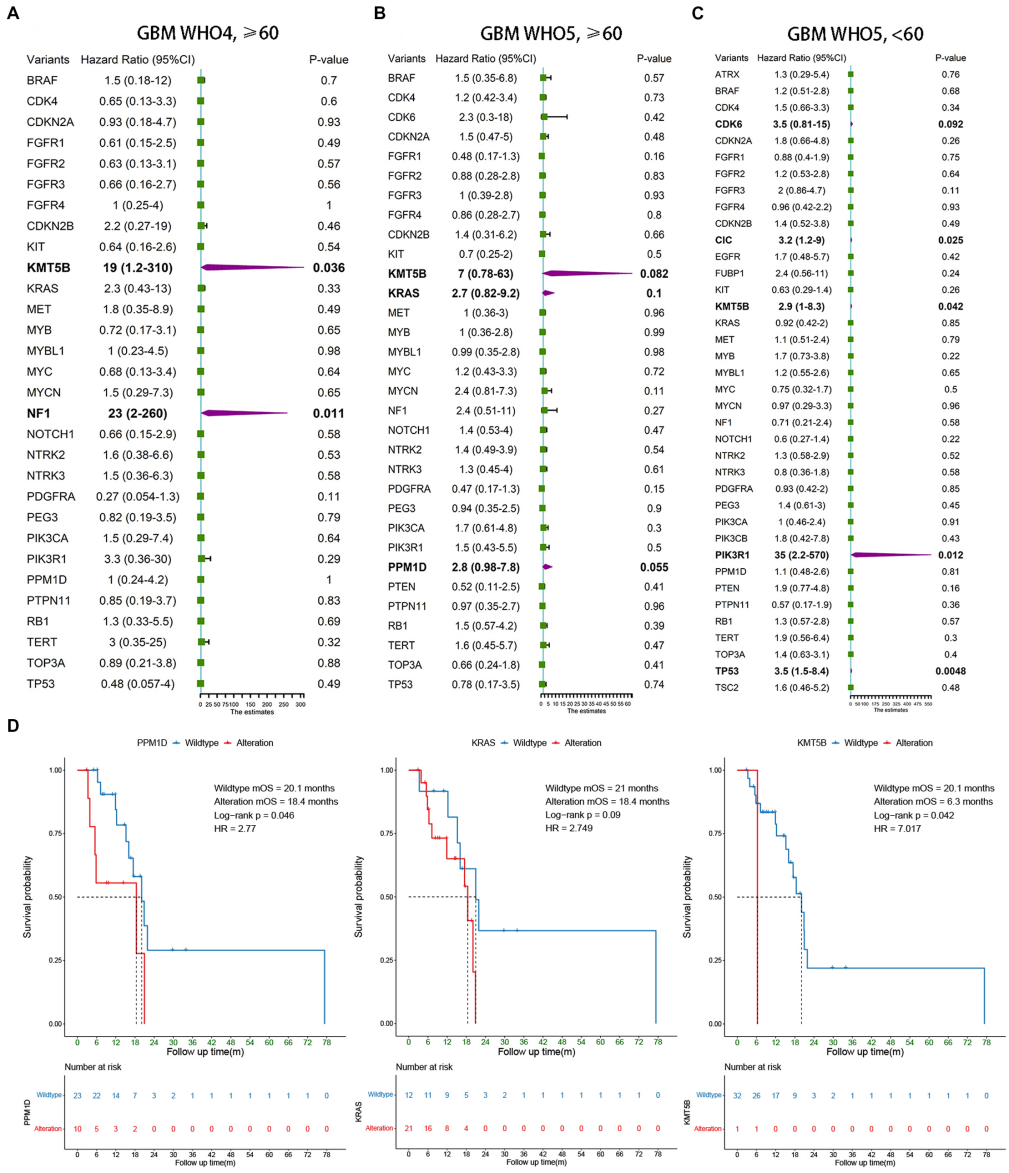
Survival analysis of younger and elderly GBM according to WHO4 and WHO5. **(A–C)** Univariate Cox regression analysis of different subgroups. Bold font represents statistical significance. **(D)** Kaplan–Meier curves were generated to illustrate the effect of the statistically significant genes in **(B)** on OS.

Univariate Cox regression was also used to analyze the three subgroups of GBM patients. In addition to the currently known *MGMT* promoter methylation status, *EGFR* amplification, and *TERT* promoter mutation, we found several other molecular markers that may have prognostic value. In elderly GBM patients under the WHO5 classification, *KMT5B* (*p* = 0.082), *KRAS* (*p* = 0.1), and *PPM1D* (*p* = 0.055) alteration were associated with poorer OS (Figure 3B), and in younger GBM patients under the WHO5 classification, *CDK6* (*p* = 0.092), *CIC* (*p* = 0.025), *KMT5B* (*p* = 0.042), *PIK3R1* (*p* = 0.012), and *TP53* (*p* = 0.0048) were predictors of prognosis (Figure 3C). After this, we analyzed elderly GBM patients under the WHO4 classification system in order to compare the differences in molecular features of elderly GBM patients under different classifications and found that *KMT5B* (*p* = 0.036) and *NF1* (*p* = 0.011) alteration were associated with poorer prognosis (Figure 3A). Thus, *PPM1D* and *KRAS* may be the most important predictors for the prognoses of elderly GBM patients under the new classification system.

To test the above conclusions further, Kaplan–Meier survival analysis was conducted for *PPM1D*, *KRAS*, and *KMT5B* (Figure 3D), and we found that *PPM1D* was an effective prognostic marker for elderly patients under the new classification (wildtype mOS 20.1 m vs. alteration mOS 18.4 m, *p* = 0.046). The role of *KMT5B* alteration was unclear from our data as only one of the 33 patients underwent such alteration, however. Perhaps expanding the sample size in the future can help to clarify the role of this gene.

The other molecules were not significantly associated with the prognosis of elderly GBM patients under the WHO5 classification, and the corresponding survival analysis is shown in Supplementary Figure S1. In Supplement Table S1 and Figure 2, we can find a high incidence of CDK6 amplification (91.3% in WHO4 elderly patients, 84.8% in WHO5 elderly patients and 83.8% in WHO5 younger patients). Moreover, CDKN2A, CDKN2B, BRAF, and FGFR2 showed a high deletion rate (Supplementary Figure S1). But these molecular alterations did not differ between groups and were a common feature of all GBM patients in this study.

## Discussion

Some clinical characteristics of WHO5 elderly patients can be summarized from the baseline results of Table 1. WHO5 elderly patients had higher Ki-67 compared to WHO5 younger patients. Most previous studies have not examined the correlation between Ki-67 and age, but one study had found that higher Ki-67 values were associated with worse OS (Liu et al., 2021), so this deserves more attention. In terms of symptoms, there was more epilepsy and intracranial hypertension in younger patients and more neurological impairment in older patients under WHO5 classification. This is in line with the results of some previous studies with larger sample sizes (Li et al., 2022). These results suggest that in elderly patients, there is a greater need to focus on the neurological aspects of the patient’s regression, especially considering that this may lead to a decrease in their cognition and quality of life, and for postoperative evaluation may also need to be more frequent.

In this study, the relationship between 60 pre-determined molecular biomarkers and the prognosis of elderly patients with GBM was examined using univariate Cox regression. By comparison with two control groups, elderly WHO4 patients and general younger WHO5 patients, we identified 2 molecular traits, *KRAS* and *PPM1D*, that were significantly associated with prognosis in elderly patients under the new WHO CNS5 classification. These results were further tested using Kaplan–Meier analysis, which reinforces the conclusion that alterations in KRAS and PPM1D lead to a significantly worse prognosis for elderly GBM patients under WHO CNS5 classification.

The molecular profile of elderly GBM patients differs considerably from that of the general younger population, and the elderly genome typically shows an unstable profile (Ferguson et al., 2016). There has already been some research on the molecular characteristics of elderly GBM patients, but given the small number of relevant studies, no study has been able to develop sufficient consensus. Current studies suggest that genes that are often up-regulated in aging GBM patients are *PRUNE2, TMEM144, SLC14A1*, and genes that are down-regulated are *H2AFY2, ENOSF1* (Bozdag et al., 2013). In conclusion, the molecular profile of GBM in elderly patients is not fully understood, but there is evidence that many genetic factors negatively affect prognosis by their interactions with other clinical features (Bruno et al., 2022).

The 2021 WHO5 classification of tumors of the central nervous system further advances the role of molecular diagnostics in the classification of CNS tumors (Louis et al., 2021). Under this classification’s new definition of GBM, the molecular profile of elderly GBM patients has changed. A recent study of a selected cohort of 212 *IDH*-wildtype GBM patients performed a more comprehensive molecular characterization of elderly GBM patients under WHO CNS5 classification and found that MGMT promoter methylation status and TERT promoter mutation status could not significantly distinguish the prognosis of elderly patients when these group of patients were analyzed alone (Fukai et al., 2020). Thus, there is still a research gap in this area, which we aim to fill with the results herein.

Our study identified *PPM1D* as a prognostic signature molecule in elderly GBM patients, but *PPM1D* has been poorly studied in GBM. Among CNS tumors, *PPM1D* has been found to be a good prognostic marker for diffuse midline gliomas (Khadka et al., 2022), younger supratentorial diffuse astrocytic tumors, and oligodendroglial tumors (Jeong et al., 2018). *PPM1D* silencing has been found to be associated with tumor sensitivity to treatment in gliomas. As an example, the combination of lentivirus-mediated silencing of *PPM1D* and temozolomide chemotherapy has been shown to eradicate malignant glioma (Wang et al., 2016), and *PPM1D* inhibitors can enhance the anti-proliferative and pro-apoptotic effects of ionizing radiation in diffuse intrinsic pontine glioma (Akamandisa et al., 2019). Given that *PPM1D* is not a clear prognostic factor in GBM, though, there are fewer studies of *PPM1D*-related therapies for GBM, and thus our findings suggest a new direction for treatment options for elderly GBM patients.

*KRAS* is one of the most important and frequently occurring oncogenic mutations in humans. Activating mutations in the *KRAS* gene are present in more than 80% of pancreatic cancers and more than 30% of colorectal cancers (Timar and Kashofer, 2020) and have been shown to be significantly associated with the poor prognosis of many cancers, such as pancreatic cancer (Buscail et al., 2020) and non-small cell lung cancer (Cai et al., 2020). However, *KRAS* is not mutated at a high rate among GBM patients and is not commonly used as a prognostic molecule in GBM treatments (Thakkar et al., 2014). Currently, sotorasib monotherapy has been approved by the Food and Drug Administration in 2021 as second-line treatment for *KRAS G12C* (Herdeis et al., 2021), but no clinical trials have been conducted with GBM. Based on the results of this study, elderly GBM patients, unlike general younger GBM patients, may be a suitable candidate for this therapy.

The generally high incidence of CDKN2A, CDKN2B, BRAF and FGFR2 deletion in the entire GBM patient population in previous GBM literature studies (Brennan et al., 2013; Jovčevska, 2018; Le Rhun et al., 2019; Senhaji et al., 2022) is consistent with our findings. However, for CDK6 amplification, we found that our statistics were too high, reaching 80–90% compared to the very low amplification in previous studies (Brennan et al., 2013). CDK6 facilitates the progression of cells through the early G1 phase of the cell cycle by forming CDK4/6-cyclin D complexes. If CDK6 amplification occurs, it will lead to cells susceptible to an uncontrollable proliferative division state, which plays an important role in promoting cancer development (Nebenfuehr et al., 2020). Therefore, subsequent investigators can start from these inconsistent data to further investigate the role of CDK6 in GBM. Overall, however, we do not go too far in this study for these findings, as these molecular alterations are not unique molecular features of WHO5 elderly patients.

Limitations of this study include potential selection bias inherent in any retrospective study, and the relatively small number of subjects compared to a standard prospective study. We included a sample of 98 elderly GBM patients from the brain tumor patient database of PUMCH, 33 of whom had molecular detection data, and this database has only been including molecular features of patients since 2016. From the results of survival analysis, patients with molecular detection data had better OS than other patients. On the one hand, this may be because patients who cooperate with doctors to complete molecular detection have better compliance; on the other hand, it may be because the sample size is small, leading to underrepresentation. Further studies with larger numbers of patients would help to solve this problem and understand the poor prognosis of GBM in the elderly more accurately. Furthermore, this study only considered altered/wildtype status without further differentiation. In the case of *KRAS*, different mutant phenotypes may have different prognosis and thus different treatment modalities (Herdeis et al., 2021), and activating mutations and deletions can also express different cancer-driven capabilities. A more refined sequencing typing with a larger sample size is therefore recommended in the future. In addition, at the mechanistic level, *PPM1D* is a relatively under-studied molecule in GBM. Although this study identified a significant prognostic role for it in elderly GBM, the underlying mechanisms by which *PPM1D* may impact the prognosis of gliomas have not been explored, which is a direction for further research.

In conclusion, this retrospective study identified *KRAS* and *PPM1D* as unique prognostic molecular features in elderly GBM patients under the new WHO CNS5 classification. In an era when molecular features are increasingly important for clinical decision-making, the study of molecular features in elderly patients with GBM should receive more attention, and our study broadens the boundaries of this field and provides a direction for subsequent studies to begin to lay the foundation for prolonging the prognosis of elderly patients with GBM.

## Data availability statement

The original contributions presented in the study are included in the article/Supplementary materials, further inquiries can be directed to the corresponding authors.

## Ethics statement

The studies involving human participants were reviewed and approved by the Regional Ethics Committee at Peking Union Medical College Hospital (S-424). The patients/participants provided their written informed consent to participate in this study. Written informed consent was obtained from the individual(s) for the publication of any potentially identifiable images or data included in this article.

## Author contributions

YanW led the research direction and was the main leader of this study. JL was responsible for the oncoplot and Univariate Cox regression analysis. YC analyzed the baseline data and was responsible for the Kaplan–Meier section. WC, HX, XG, YS, YueW, TL, LY, DL, TY, YuW, and WM contributed to the clinical, radiological, and tumor pathological data collection. All authors contributed to the article and approved the submitted version.

## Funding

This work was funded by the Beijing Municipal Natural Science Foundation (7202150) and the National High Level Hospital Clinical Research Funding (2022-PUMCH-A-019) for YuW, and by the National High Level Hospital Clinical Research Funding (2022-PUMCH-B-113), the Tsinghua University-Peking Union Medical College Hospital Initiative Scientific Research Program (2019ZLH101) and the Beijing Municipal Natural Science Foundation (19JCZDJC64200[Z]) for WM.

## Conflict of interest

The authors declare that the research was conducted in the absence of any commercial or financial relationships that could be construed as a potential conflict of interest.

## Publisher’s note

All claims expressed in this article are solely those of the authors and do not necessarily represent those of their affiliated organizations, or those of the publisher, the editors and the reviewers. Any product that may be evaluated in this article, or claim that may be made by its manufacturer, is not guaranteed or endorsed by the publisher.

## Abbreviations

GBM: glioblastoma
WHO5: The WHO Classification of Central Nervous System Tumors
PUMCH: Peking Union Medical College Hospital
BMI: body mass index
KPS: Karnofsky performance status
OS: overall survival
FFPE: formalin-fixed paraffin-embedded
MRI: magnetic resonance imaging
