# Supplementary material for: Characteristic analysis and identification of novel molecular biomarkers in elderly glioblastoma patients using the 2021 WHO Classification of Central Nervous System Tumors
Source: Front Neurosci. 2023 Jun 9;17:1165823. doi: 10.3389/fnins.2023.1165823 (PMC10288210; doi:10.3389/fnins.2023.1165823)
Supplement: Supplementary file 1 [file Table_1.DOCX]

|  | GBM WHO4, ≥60 (n=23) | | |  | GBM WHO5, ≥60 (n=33) | | |  | GBM WHO5, <60 (n=37) | | |
| --- | --- | --- | --- | --- | --- | --- | --- | --- | --- | --- | --- |
|  | **Mutation** | **Deletion** | **Amplification** |  | **Mutation** | **Deletion** | **Amplification** |  | **Mutation** | **Deletion** | **Amplification** |
| *ACVR1* | 0, 0.0% | 0, 0.0% | 0, 0.0% |  | 0, 0.0% | 0, 0.0% | 0, 0.0% |  | 0, 0.0% | 0, 0.0% | 0, 0.0% |
| *ATRX* | 1, 4.3% | 0, 0.0% | 0, 0.0% |  | 0, 0.0% | 0, 0.0% | 0, 0.0% |  | 3, 8.1% | 0, 0.0% | 0, 0.0% |
| *BCOR* | 1, 4.3% | 0, 0.0% | 0, 0.0% |  | 2, 6.1% | 0, 0.0% | 0, 0.0% |  | 0, 0.0% | 0, 0.0% | 0, 0.0% |
| *BRAF* | 8, 34.8% | 1, 4.3% | 9, 39.1% |  | 10, 30.3% | 2, 6.1% | 14, 42.4% |  | 14, 37.8% | 0, 0.0% | 10, 27.0% |
| *CDK4* | 5, 21.7% | 14, 60.9% | 1, 4.3% |  | 9, 27.3% | 13, 39.4% | 3, 9.1% |  | 4, 10.8% | 8, 21.6% | 7, 18.9% |
| *CDK6* | 1, 4.3% | 0, 0.0% | 21, 91.3% |  | 2, 6.1% | 0, 0.0% | 28, 84.8% |  | 1, 2.7% | 0, 0.0% | 31, 83.8% |
| *CDKN2A* | 1, 4.3% | 16, 69.6% | 3, 13.0% |  | 2, 6.1% | 22, 66.7% | 3, 9.1% |  | 1, 2.7% | 22, 59.5% | 6, 16.2% |
| *CDKN2B* | 1, 4.3% | 16, 69.6% | 3, 13.0% |  | 3, 9.1% | 22, 66.7% | 4, 12.1% |  | 2, 5.4% | 21, 56.8% | 7, 18.9% |
| *CIC* | 1, 4.3% | 0, 0.0% | 0, 0.0% |  | 1, 3.0% | 0, 0.0% | 0, 0.0% |  | 5, 13.5% | 0, 0.0% | 0, 0.0% |
| *EGFR* | 10, 43.5% | 0, 0.0% | 11, 47.8% |  | 14, 42.4% | 1, 3.0% | 17, 51.5% |  | 14, 37.8% | 1, 2.7% | 17, 45.9% |
| *FBXW7* | 0, 0.0% | 0, 0.0% | 0, 0.0% |  | 0, 0.0% | 0, 0.0% | 0, 0.0% |  | 1, 2.7% | 0, 0.0% | 0, 0.0% |
| *FGFR1* | 1, 4.3% | 13, 56.5% | 1, 4.3% |  | 1, 3.0% | 16, 48.5% | 2, 6.1% |  | 4, 10.8% | 11, 29.7% | 2, 5.4% |
| *FGFR2* | 2, 8.7% | 17, 73.9% | 0, 0.0% |  | 4, 12.1% | 22, 66.7% | 0, 0.0% |  | 2, 5.4% | 23, 62.2% | 0, 0.0% |
| *FGFR3* | 0, 0.0% | 13, 56.5% | 1, 4.3% |  | 1, 3.0% | 17, 51.5% | 1, 3.0% |  | 2, 5.4% | 13, 35.1% | 0, 0.0% |
| *FGFR4* | 2, 8.7% | 10, 43.5% | 0, 0.0% |  | 2, 6.1% | 10, 30.3% | 0, 0.0% |  | 4, 10.8% | 11, 29.7% | 1, 2.7% |
| *FUBP1* | 1, 4.3% | 0, 0.0% | 0, 0.0% |  | 1, 3.0% | 0, 0.0% | 0, 0.0% |  | 2, 5.4% | 0, 0.0% | 0, 0.0% |
| *H3F3A* | 0, 0.0% | 0, 0.0% | 0, 0.0% |  | 0, 0.0% | 0, 0.0% | 0, 0.0% |  | 0, 0.0% | 0, 0.0% | 0, 0.0% |
| *HIST1H3B* | 0, 0.0% | 0, 0.0% | 0, 0.0% |  | 0, 0.0% | 0, 0.0% | 0, 0.0% |  | 0, 0.0% | 0, 0.0% | 0, 0.0% |
| *HIST1H3C* | 0, 0.0% | 0, 0.0% | 0, 0.0% |  | 0, 0.0% | 0, 0.0% | 0, 0.0% |  | 0, 0.0% | 0, 0.0% | 0, 0.0% |
| *IDH1* | 1, 4.3% | 0, 0.0% | 0, 0.0% |  | 0, 0.0% | 0, 0.0% | 0, 0.0% |  | 0, 0.0% | 0, 0.0% | 0, 0.0% |
| *IDH2* | 0, 0.0% | 0, 0.0% | 0, 0.0% |  | 0, 0.0% | 0, 0.0% | 0, 0.0% |  | 0, 0.0% | 0, 0.0% | 0, 0.0% |
| *KIT* | 5, 21.7% | 0, 0.0% | 9, 39.1% |  | 7, 21.2% | 1, 3.0% | 9, 27.3% |  | 3, 8.1% | 1, 2.7% | 16, 43.2% |
| *KMT5B* | 1, 4.3% | 0, 0.0% | 0, 0.0% |  | 1, 3.0% | 0, 0.0% | 0, 0.0% |  | 5, 13.5% | 0, 0.0% | 0, 0.0% |
| *KRAS* | 4, 17.4% | 5, 21.7% | 8, 34.8% |  | 7, 21.2% | 5, 15.2% | 9, 27.3% |  | 3, 8.1% | 2, 5.4% | 11, 29.7% |
| *MAP2K1* | 0, 0.0% | 0, 0.0% | 0, 0.0% |  | 0, 0.0% | 0, 0.0% | 0, 0.0% |  | 0, 0.0% | 0, 0.0% | 0, 0.0% |
| *MET* | 0, 0.0% | 0, 0.0% | 15, 65.2% |  | 3, 9.1% | 0, 0.0% | 19, 57.6% |  | 5, 13.5% | 0, 0.0% | 17, 45.9% |
| *MYB* | 3, 13.0% | 1, 4.3% | 14, 60.9% |  | 4, 12.1% | 4, 12.1% | 15, 45.5% |  | 6, 16.2% | 9, 24.3% | 7, 18.9% |
| *MYBL1* | 1, 4.3% | 0, 0.0% | 12, 52.2% |  | 1, 3.0% | 1, 3.0% | 14, 42.4% |  | 3, 8.1% | 0, 0.0% | 12, 32.4% |
| *MYC* | 3, 13.0% | 4, 17.4% | 2, 8.7% |  | 4, 12.1% | 6, 18.2% | 3, 9.1% |  | 4, 10.8% | 5, 13.5% | 3, 8.1% |
| *MYCN* | 4, 17.4% | 1, 4.3% | 0, 0.0% |  | 4, 12.1% | 2, 6.1% | 3, 9.1% |  | 0, 0.0% | 2, 5.4% | 3, 8.1% |
| *NF1* | 2, 8.7% | 0, 0.0% | 0, 0.0% |  | 3, 9.1% | 0, 0.0% | 0, 0.0% |  | 5, 13.5% | 0, 0.0% | 0, 0.0% |
| *NOTCH1* | 6, 26.1% | 2, 8.7% | 4, 17.4% |  | 5, 15.2% | 3, 9.1% | 7, 21.2% |  | 3, 8.1% | 5, 13.5% | 7, 18.9% |
| *NRAS* | 0, 0.0% | 0, 0.0% | 0, 0.0% |  | 0, 0.0% | 0, 0.0% | 0, 0.0% |  | 0, 0.0% | 0, 0.0% | 0, 0.0% |
| *NTRK2* | 4, 17.4% | 1, 4.3% | 8, 34.8% |  | 5, 15.2% | 2, 6.1% | 11, 33.3% |  | 3, 8.1% | 5, 13.5% | 14, 37.8% |
| *NTRK3* | 8, 34.8% | 1, 4.3% | 4, 17.4% |  | 10, 30.3% | 1, 3.0% | 5, 15.2% |  | 6, 16.2% | 3, 8.1% | 8, 21.6% |
| *PDGFRA* | 7, 30.4% | 1, 4.3% | 5, 21.7% |  | 13, 39.4% | 1, 3.0% | 5, 15.2% |  | 10, 27.0% | 1, 2.7% | 10, 27.0% |
| *PEG3* | 2, 8.7% | 1, 4.3% | 12, 52.2% |  | 3, 9.1% | 1, 3.0% | 14, 42.4% |  | 4, 10.8% | 1, 2.7% | 16, 43.2% |
| *PIK3CA* | 1, 4.3% | 1, 4.3% | 12, 52.2% |  | 3, 9.1% | 1, 3.0% | 15, 45.5% |  | 1, 2.7% | 0, 0.0% | 23, 62.2% |
| *PIK3CB* | 0, 0.0% | 0, 0.0% | 0, 0.0% |  | 0, 0.0% | 0, 0.0% | 0, 0.0% |  | 2, 5.4% | 0, 0.0% | 0, 0.0% |
| *PIK3R1* | 1, 4.3% | 0, 0.0% | 0, 0.0% |  | 3, 9.1% | 0, 0.0% | 0, 0.0% |  | 1, 2.7% | 0, 0.0% | 0, 0.0% |
| *PPM1D* | 3, 13.0% | 0, 0.0% | 5, 21.7% |  | 4, 12.1% | 0, 0.0% | 6, 18.2% |  | 4, 10.8% | 1, 2.7% | 6, 16.2% |
| *PTEN* | 5, 21.7% | 13, 56.5% | 0, 0.0% |  | 5, 15.2% | 19, 57.6% | 3, 9.1% |  | 9, 24.3% | 16, 43.2% | 1, 2.7% |
| *PTPN11* | 3, 13.0% | 9, 39.1% | 1, 4.3% |  | 6, 18.2% | 11, 33.3% | 2, 6.1% |  | 0, 0.0% | 6, 16.2% | 1, 2.7% |
| *RB1* | 2, 8.7% | 3, 13.0% | 8, 34.8% |  | 4, 12.1% | 4, 12.1% | 10, 30.3% |  | 4, 10.8% | 8, 21.6% | 9, 24.3% |
| *SMARCA4* | 0, 0.0% | 0, 0.0% | 0, 0.0% |  | 1, 3.0% | 0, 0.0% | 0, 0.0% |  | 1, 2.7% | 0, 0.0% | 0, 0.0% |
| *SMARCB1* | 0, 0.0% | 0, 0.0% | 0, 0.0% |  | 0, 0.0% | 0, 0.0% | 0, 0.0% |  | 0, 0.0% | 0, 0.0% | 0, 0.0% |
| *TERT* | 19, 82.6% | 0, 0.0% | 0, 0.0% |  | 26, 78.8% | 0, 0.0% | 0, 0.0% |  | 31, 83.8% | 0, 0.0% | 0, 0.0% |
| *TOP3A* | 7, 30.4% | 10, 43.5% | 0, 0.0% |  | 8, 24.2% | 12, 36.4% | 2, 6.1% |  | 12, 32.4% | 4, 10.8% | 2, 5.4% |
| *TP53* | 3, 13.0% | 0, 0.0% | 0, 0.0% |  | 3, 9.1% | 0, 0.0% | 0, 0.0% |  | 9, 24.3% | 0, 0.0% | 0, 0.0% |
| *TSC1* | 0, 0.0% | 0, 0.0% | 0, 0.0% |  | 0, 0.0% | 0, 0.0% | 0, 0.0% |  | 0, 0.0% | 0, 0.0% | 0, 0.0% |
| *TSC2* | 0, 0.0% | 0, 0.0% | 0, 0.0% |  | 0, 0.0% | 0, 0.0% | 0, 0.0% |  | 3, 8.1% | 0, 0.0% | 0, 0.0% |
| *YAP1* | 0, 0.0% | 0, 0.0% | 0, 0.0% |  | 0, 0.0% | 0, 0.0% | 0, 0.0% |  | 0, 0.0% | 0, 0.0% | 0, 0.0% |
| chr1p | 19, 82.6% | 1, 4.3% | 3, 13.0% |  | 27, 81.8% | 1, 3.0% | 5, 15.2% |  | 26, 70.3% | 2, 5.4% | 8, 21.6% |
| chr7p | 2, 8.7% | 0, 0.0% | 21, 91.3% |  | 3, 9.1% | 0, 0.0% | 30, 90.9% |  | 5, 13.5% | 1, 2.7% | 30, 81.1% |
| chr7q | 5, 21.7% | 0, 0.0% | 18, 78.3% |  | 7, 21.2% | 0, 0.0% | 26, 78.8% |  | 12, 32.4% | 0, 0.0% | 23, 62.2% |
| chr9p | 4, 17.4% | 15, 65.2% | 4, 17.4% |  | 8, 24.2% | 21, 63.6% | 4, 12.1% |  | 12, 32.4% | 19, 51.4% | 5, 13.5% |
| chr10p | 7, 30.4% | 15, 65.2% | 1, 4.3% |  | 11, 33.3% | 22, 66.7% | 0, 0.0% |  | 6, 16.2% | 29, 78.4% | 1, 2.7% |
| chr10q | 3, 13.0% | 19, 82.6% | 1, 4.3% |  | 7, 21.2% | 25, 75.8% | 1, 3.0% |  | 8, 21.6% | 28, 75.7% | 0, 0.0% |
| chr17 | 18, 78.3% | 1, 4.3% | 3, 13.0% |  | 25, 75.8% | 1, 3.0% | 5, 15.2% |  | 28, 75.7% | 3, 8.1% | 3, 8.1% |
| chr19q | 9, 39.1% | 3, 13.0% | 9, 39.1% |  | 16, 48.5% | 3, 9.1% | 10, 30.3% |  | 19, 51.4% | 2, 5.4% | 9, 24.3% |

Supplementary Table 1. Molecular alterations of different subtypes of GBM classified by the 4th and 5th edition of the WHO classification of CNS tumors
