# Supplementary material for: Characteristic analysis and identification of novel molecular biomarkers in elderly glioblastoma patients using the 2021 WHO Classification of Central Nervous System Tumors
Source: Front Neurosci. 2023 Jun 9;17:1165823. doi: 10.3389/fnins.2023.1165823 (PMC10288210; doi:10.3389/fnins.2023.1165823)
Supplement: Supplementary file 2 [file Data_Sheet_1.PDF]

Supplement Figure1. Kaplan–Meier curves showing effects of alterations of remaining genes on the overall survival of aged WHO5 GBM patients. Parameters used in this section were shown as follows.

(1) BRAF 1
(2) CDK4 2
(3) CDK6 3
(4) CDKN2A..4
(5) CDKN2B..5
(6) FGFR1..6
(7) FGFR2..7
(8) FGFR3..8
(9) FGFR4..9
(10) KIT 10
(11) MET..11
(12) MYB..12
(13) MYBL1 13
(14) MYC..14
(15) MYCN..15
(16) NF1 16
(17) NOTCH1..17
(18) NTRK2..18
(19) NTRK3..19
(20) PDGFRA..20
(21) PEG3 21
(22) PIK3CA..22
(23) PIK3R1..23
(24) PTEN..24
(25) PTPN11 25
(26) RB1..26
(27) TERT 27
(28) TOP3A 28
(29) TP53 29

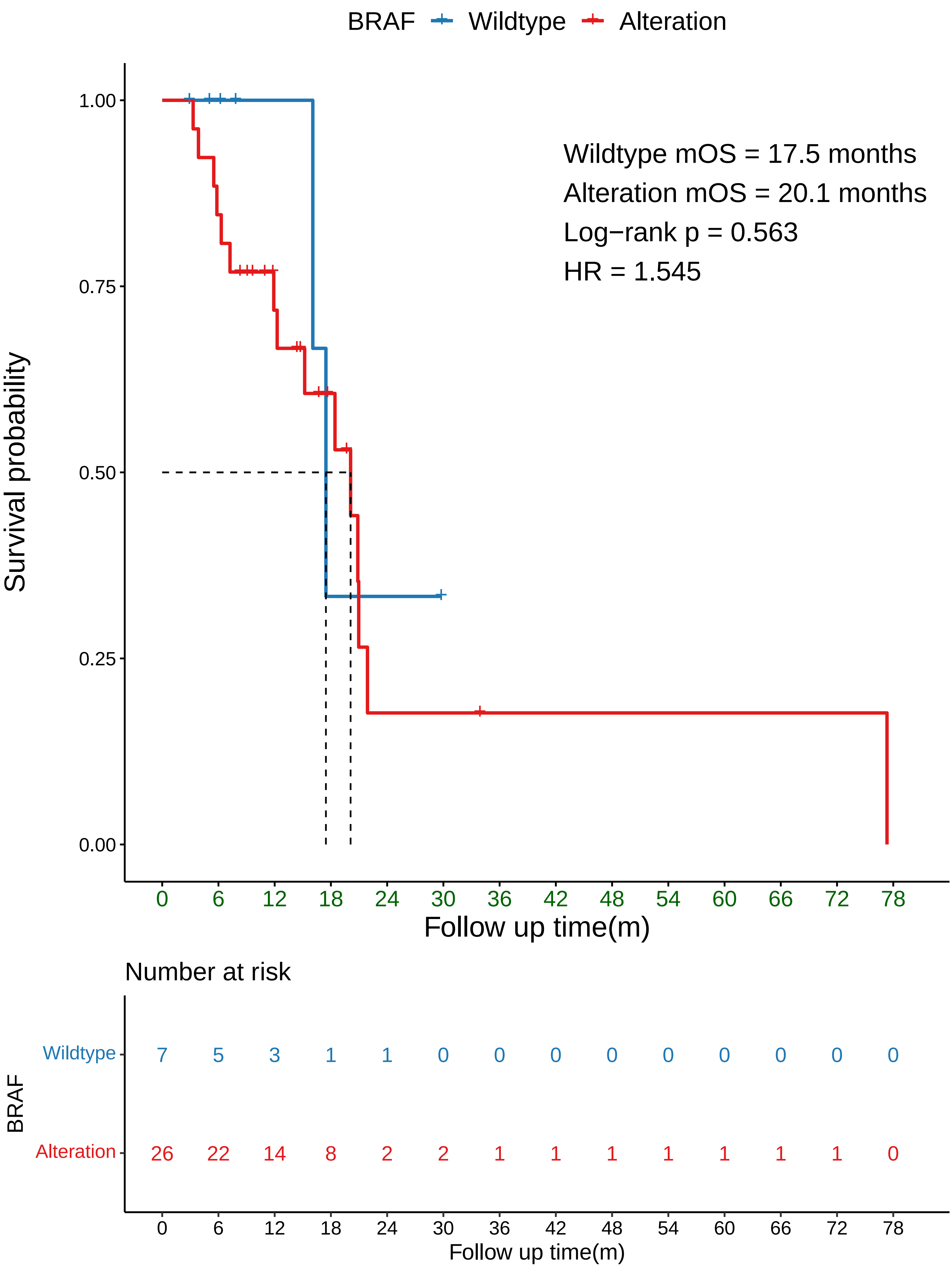

BRAF
Wildtype
Alteration
Wildtype mOS = 17.5 months
Alteration mOS = 20.1 months
Log−rank p = 0.563
HR = 1.545
Survival probability
1.00
0.75
0.50
0.25
0.00
0 6 12 18 24 30 36 42 48 54 60 66 72 78
Follow up time(m)
Number at risk
BRAF
Wildtype 7 5 3 1 1 0 0 0 0 0 0 0 0 0
Alteration 26 22 14 8 2 2 1 1 1 1 1 1 1 0
0 6 12 18 24 30 36 42 48 54 60 66 72 78
Follow up time(m)

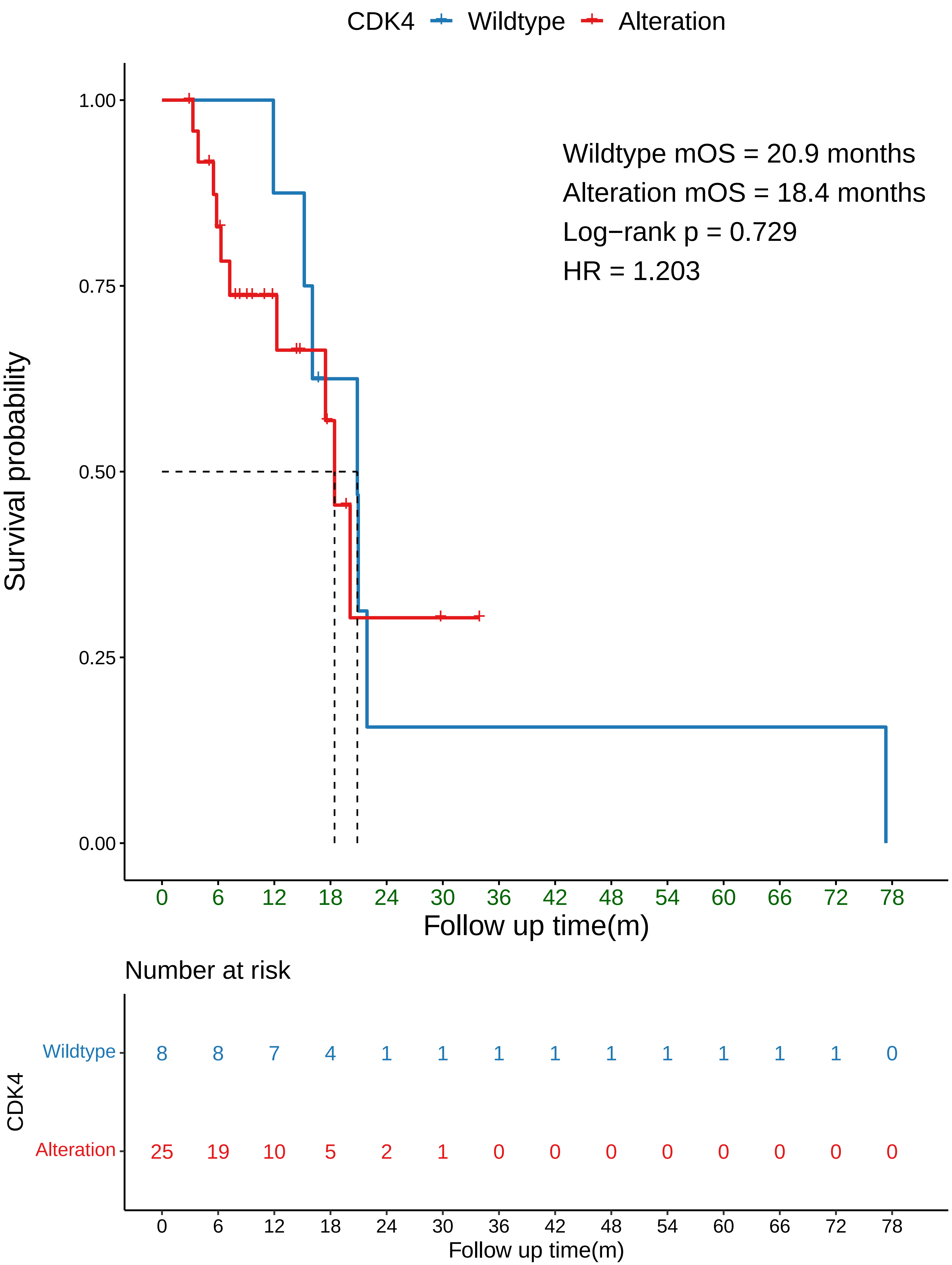

CDK4
Wildtype
Alteration
Wildtype mOS = 20.9 months
Alteration mOS = 18.4 months
Log−rank p = 0.729
HR = 1.203
Survival probability
1.00
0.75
0.50
0.25
0.00
0 6 12 18 24 30 36 42 48 54 60 66 72 78
Follow up time(m)
Number at risk
Wildtype 8 8 7 4 1 1 1 1 1 1 1 1 1 0
Alteration 25 19 10 5 2 1 0 0 0 0 0 0 0 0
0 6 12 18 24 30 36 42 48 54 60 66 72 78
Follow up time(m)

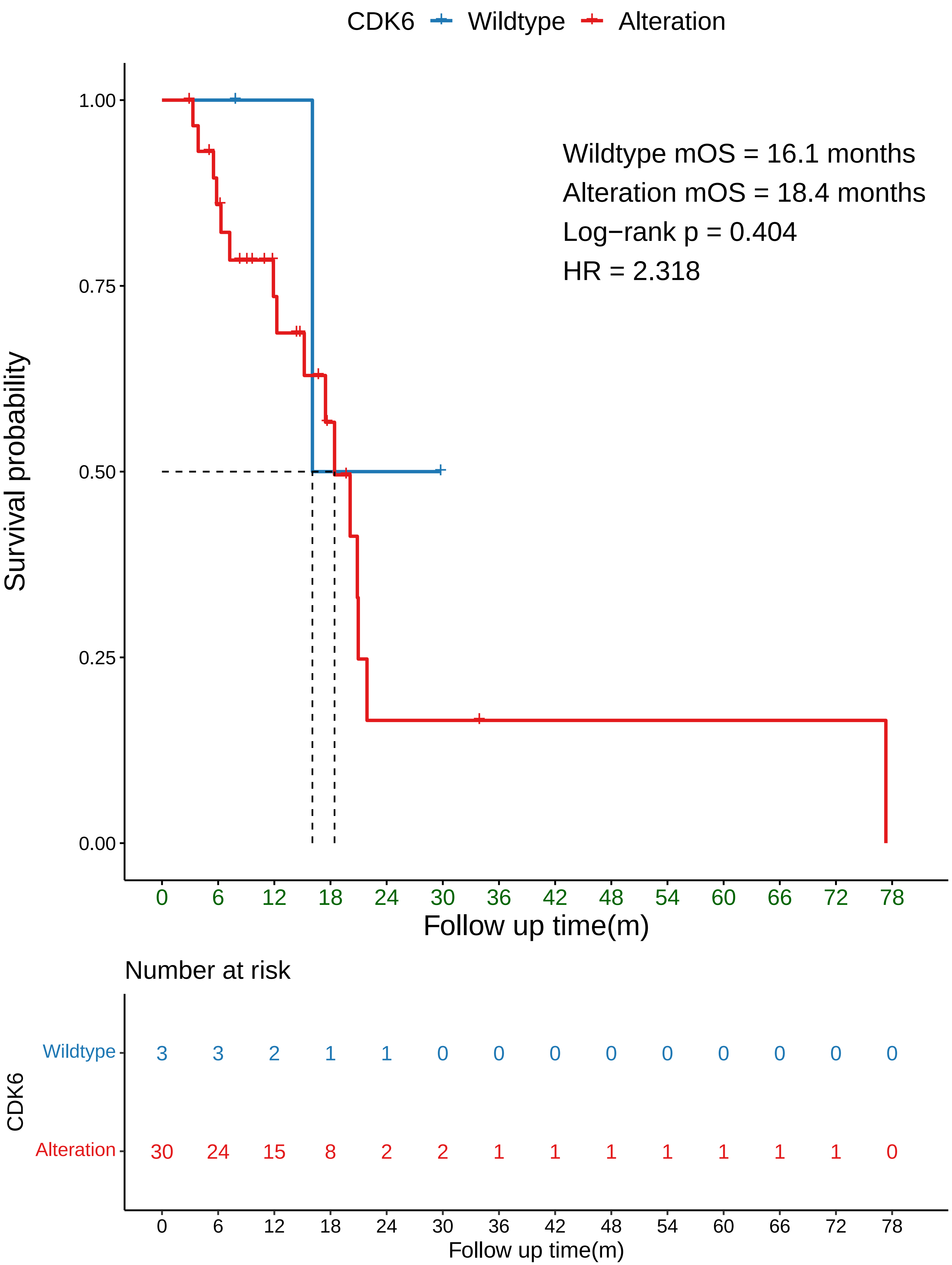

CDK6
Wildtype
Alteration
Wildtype mOS = 16.1 months
Alteration mOS = 18.4 months
Log−rank p = 0.404
HR = 2.318
Survival probability
1.00
0.75
0.50
0.25
0.00
0 6 12 18 24 30 36 42 48 54 60 66 72 78
Follow up time(m)
Number at risk
CDK6
Wildtype 3 3 2 1 1 0 0 0 0 0 0 0 0 0
Alteration 30 24 15 8 2 2 1 1 1 1 1 1 1 0
0 6 12 18 24 30 36 42 48 54 60 66 72 78
Follow up time(m)

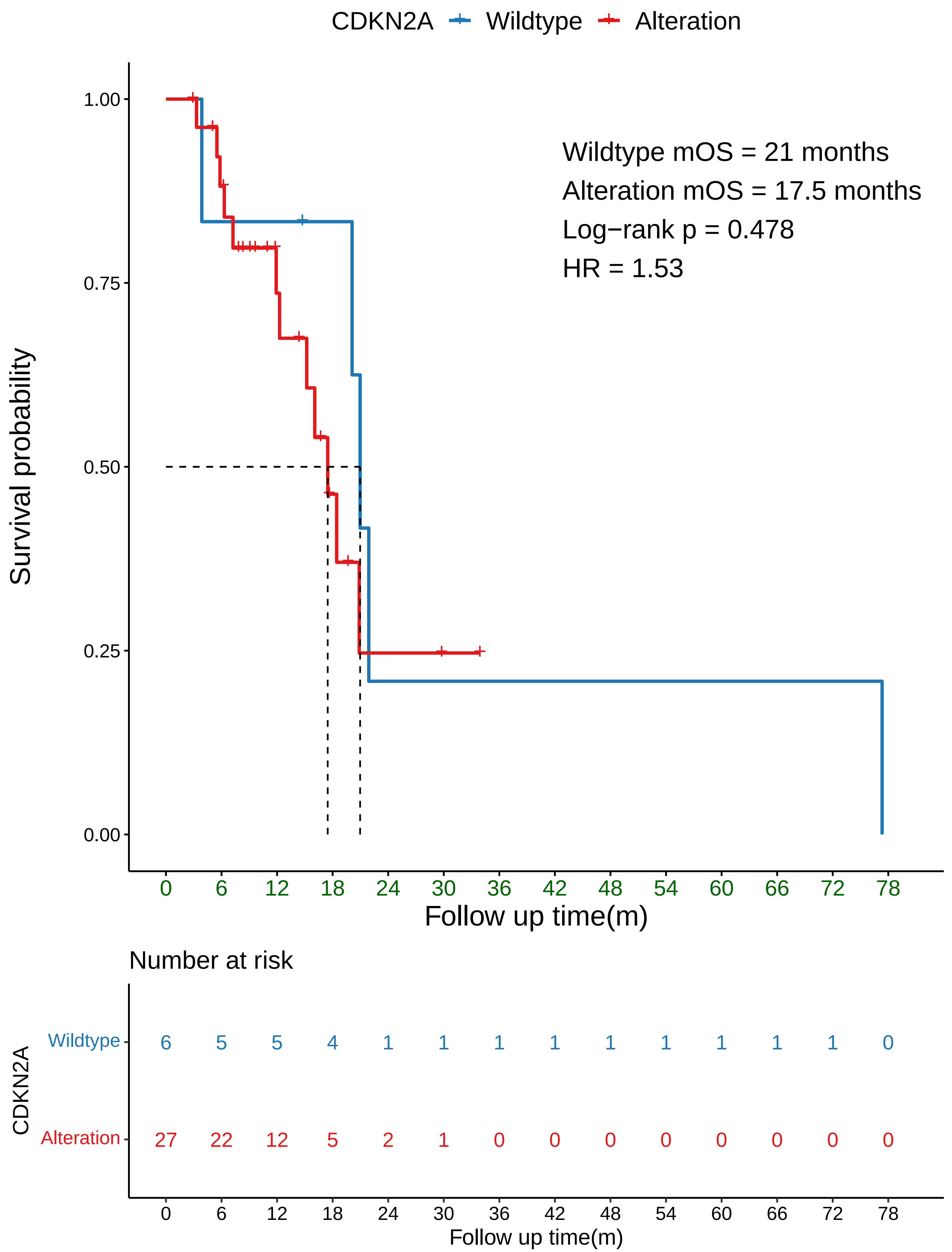

CDKN2A
Wildtype
Alteration
Wildtype mOS = 21 months
Alteration mOS = 17.5 months
Log−rank p = 0.478
HR = 1.53
Survival probability
1.00
0.75
0.50
0.25
0.00
0 6 12 18 24 30 36 42 48 54 60 66 72 78
Follow up time(m)
Number at risk
CDKN2A
Wildtype 6 5 5 4 1 1 1 1 1 1 1 1 1 0
Alteration 27 22 12 5 2 1 0 0 0 0 0 0 0 0
0 6 12 18 24 30 36 42 48 54 60 66 72 78
Follow up time(m)

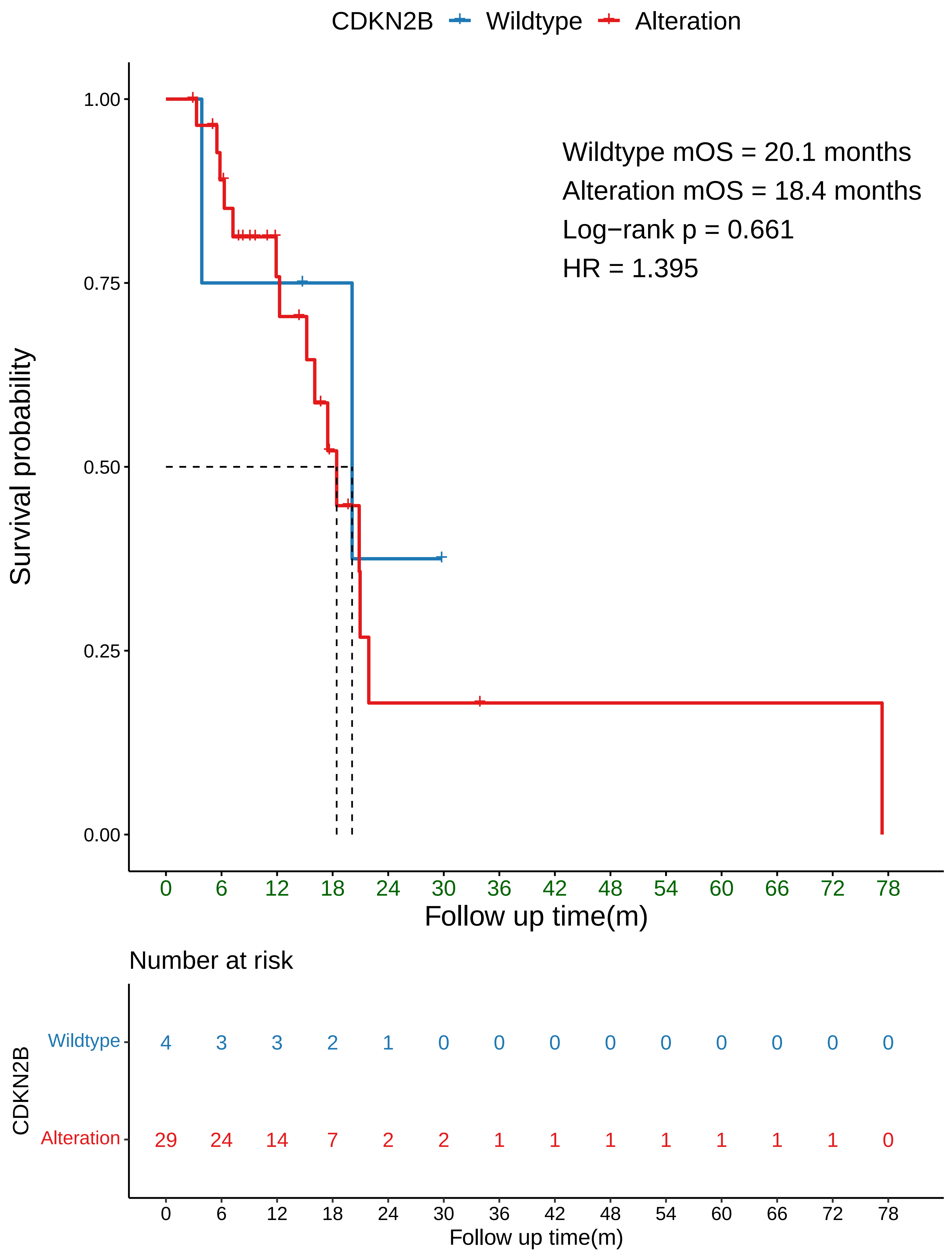

CDKN2B
Wildtype
Alteration
Wildtype mOS = 20.1 months
Alteration mOS = 18.4 months
Log−rank p = 0.661
HR = 1.395
Survival probability
1.00
0.75
0.50
0.25
0.00
0
6
12
18
24
30
36
42
48
54
60
66
72
78
Follow up time(m)
Number at risk
CDKN2B
Wildtype 4 3 3 2 1 0 0 0 0 0 0 0 0 0
Alteration 29 24 14 7 2 2 1 1 1 1 1 1 1 0
0 6 12 18 24 30 36 42 48 54 60 66 72 78
Follow up time(m)

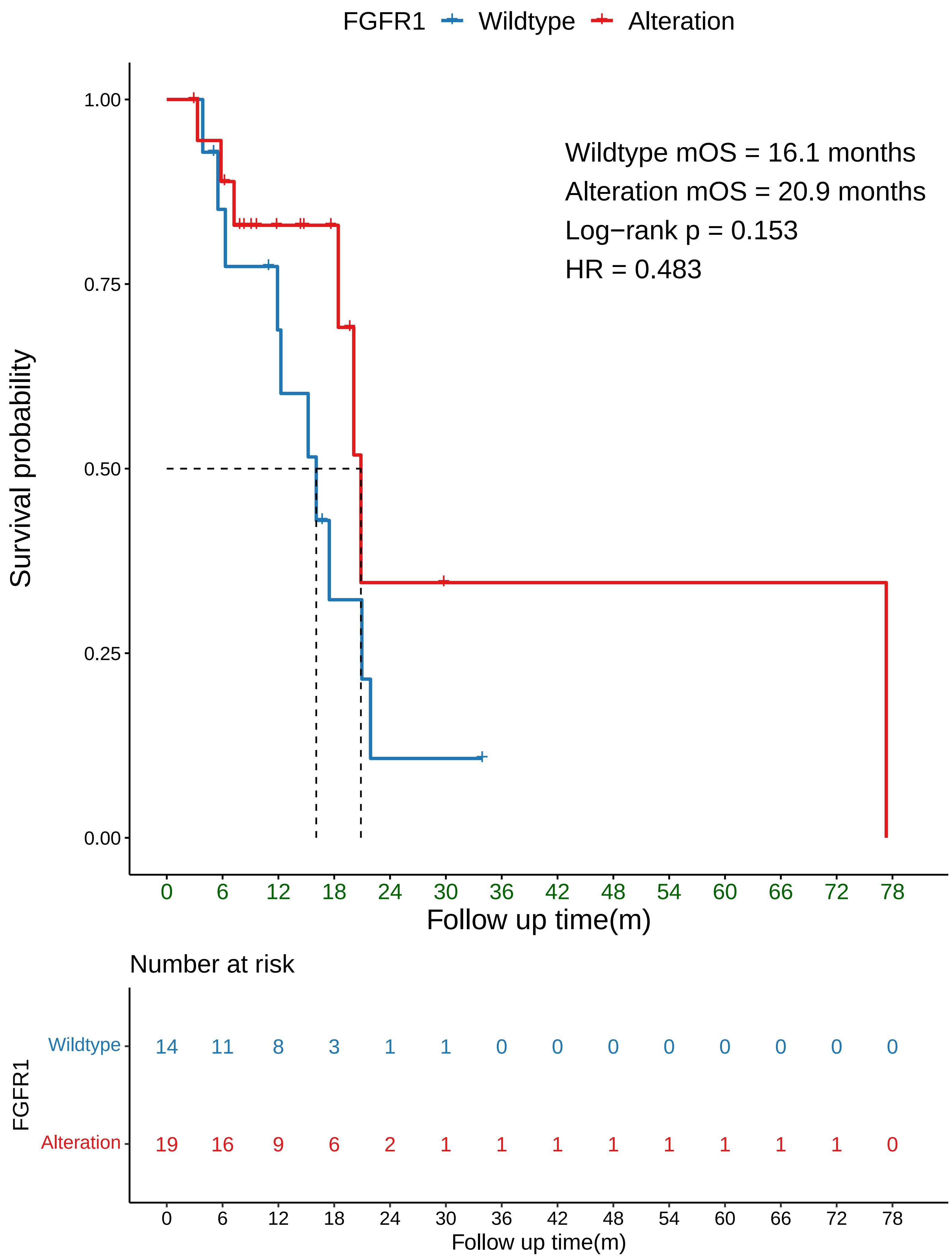
FGFR1 Wildtype Alteration
Wildtype mOS = 16.1 months
Alteration mOS = 20.9 months
Log−rank p = 0.153
HR = 0.483
Survival probability
1.00
0.75
0.50
0.25
0.00
0 6 12 18 24 30 36 42 48 54 60 66 72 78
Follow up time(m)
Number at risk
FGFR1
Wildtype 14 11 8 3 1 1 0 0 0 0 0 0 0 0
Alteration 19 16 9 6 2 1 1 1 1 1 1 1 1 0
0 6 12 18 24 30 36 42 48 54 60 66 72 78
Follow up time(m)

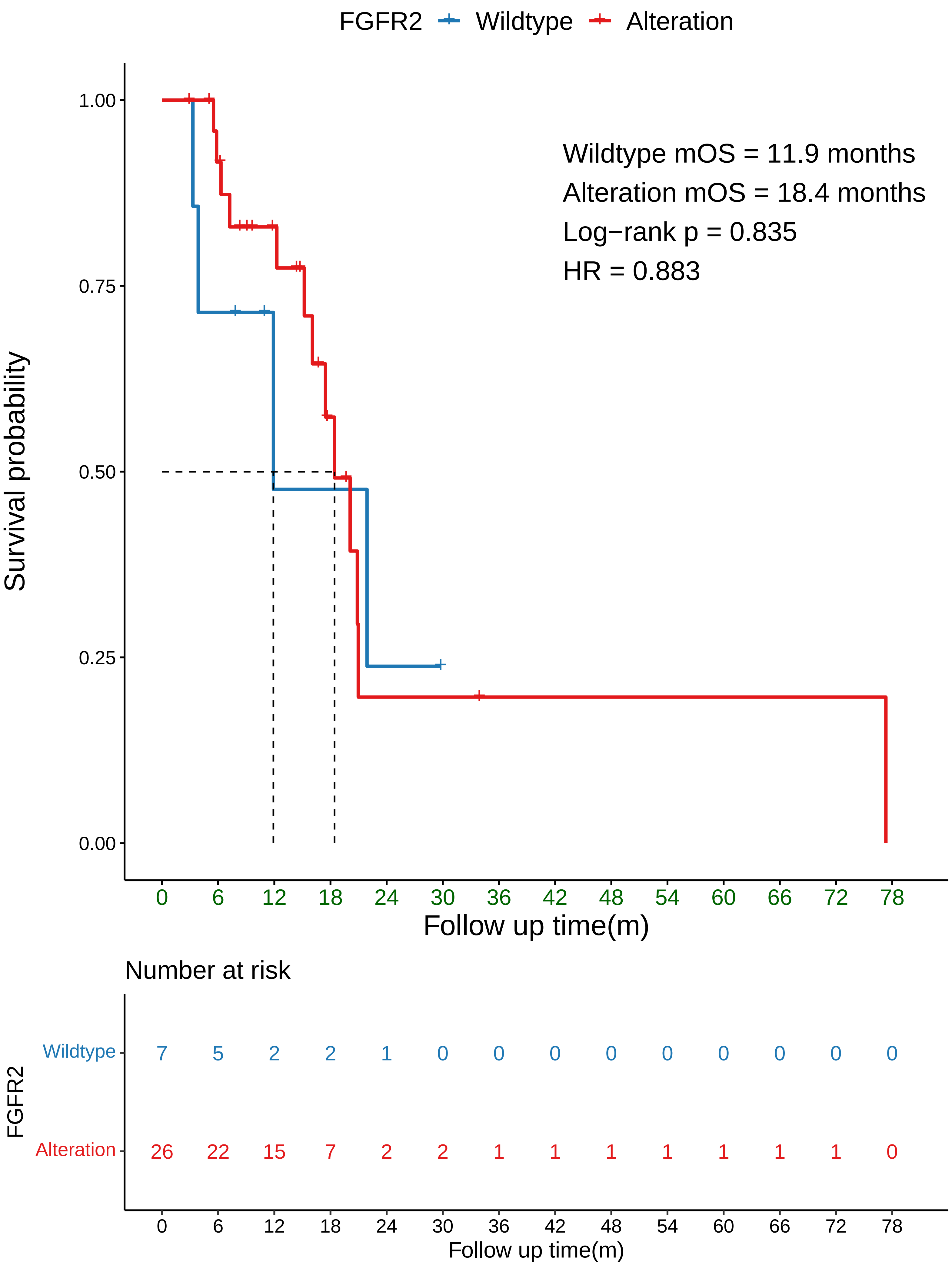

FGFR2
Wildtype
Alteration
Wildtype mOS = 11.9 months
Alteration mOS = 18.4 months
Log−rank p = 0.835
HR = 0.883
Survival probability
1.00
0.75
0.50
0.25
0.00
0 6 12 18 24 30 36 42 48 54 60 66 72 78
Follow up time(m)
Number at risk
FGFR2
Wildtype 7 5 2 2 1 0 0 0 0 0 0 0 0 0
Alteration 26 22 15 7 2 2 1 1 1 1 1 1 1 0
0 6 12 18 24 30 36 42 48 54 60 66 72 78
Follow up time(m)

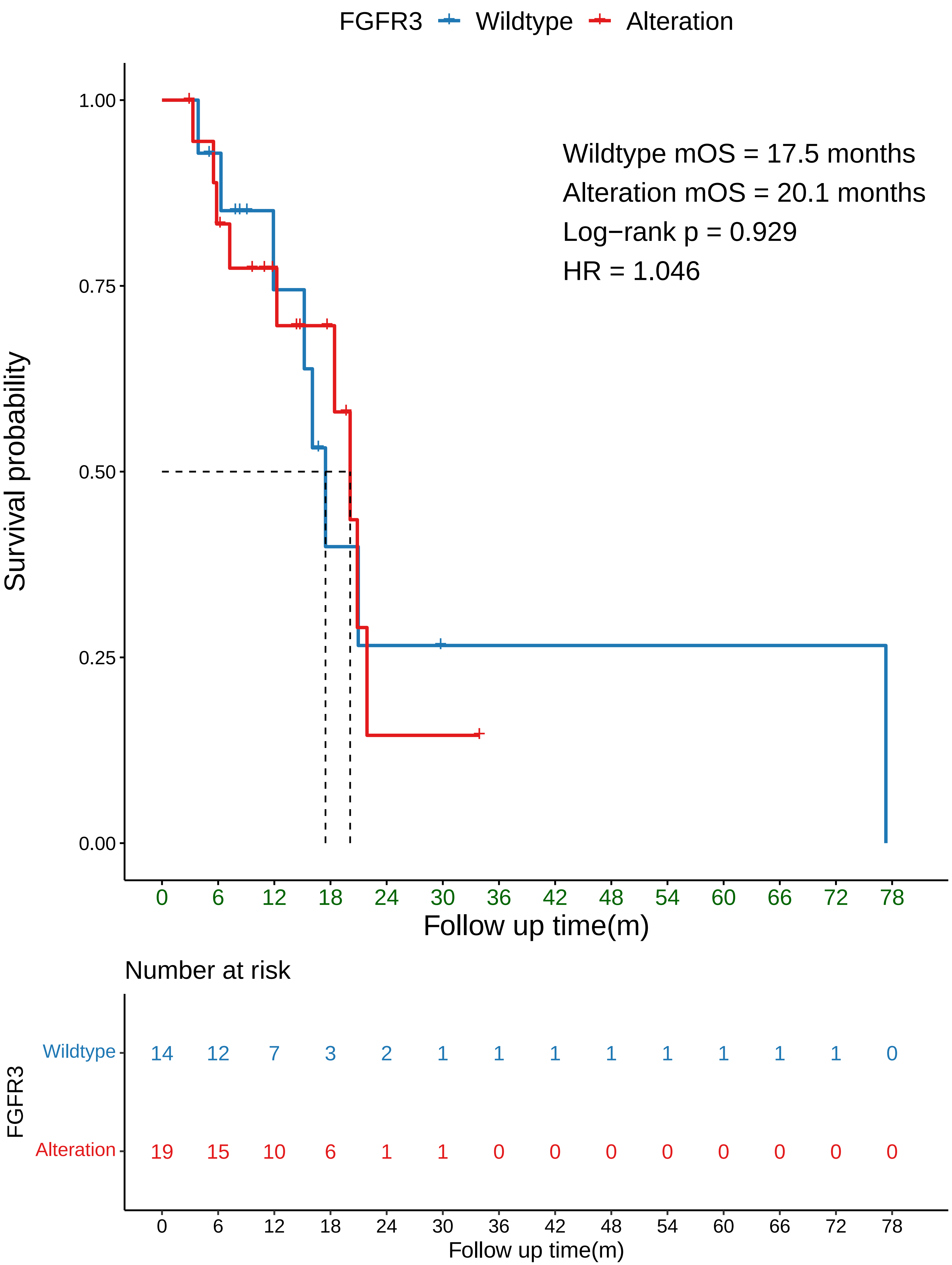

FGFR3
Wildtype
Alteration
Wildtype mOS = 17.5 months
Alteration mOS = 20.1 months
Log−rank p = 0.929
HR = 1.046
Survival probability
1.00
0.75
0.50
0.25
0.00
0 6 12 18 24 30 36 42 48 54 60 66 72 78
Follow up time(m)
Number at risk
FGFR3
Wildtype 14 12 7 3 2 1 1 1 1 1 1 1 1 0
Alteration 19 15 10 6 1 1 0 0 0 0 0 0 0 0
0 6 12 18 24 30 36 42 48 54 60 66 72 78
Follow up time(m)

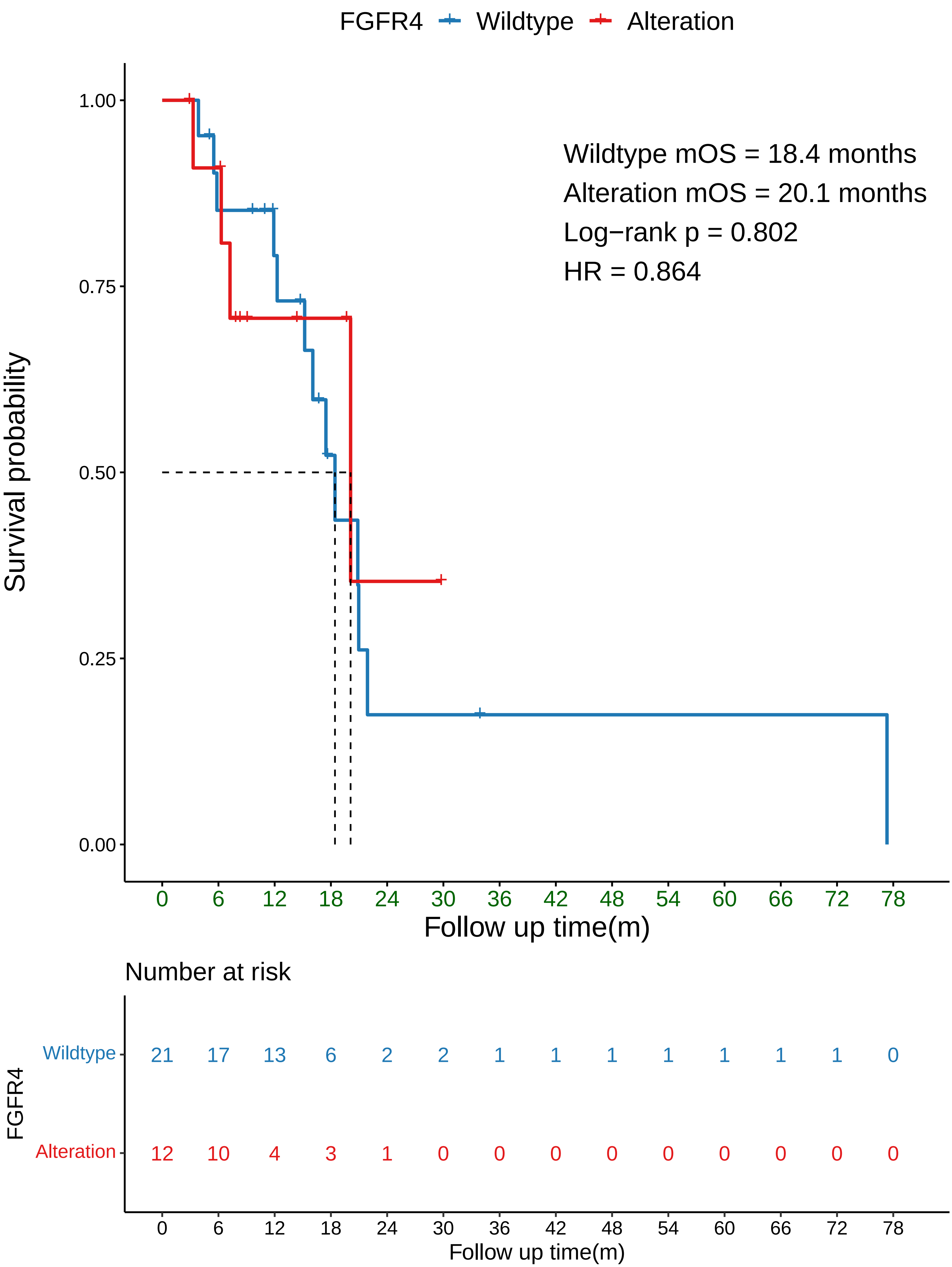

FGFR4
Wildtype
Alteration
Wildtype mOS = 18.4 months
Alteration mOS = 20.1 months
Log−rank p = 0.802
HR = 0.864
Survival probability
1.00
0.75
0.50
0.25
0.00
0 6 12 18 24 30 36 42 48 54 60 66 72 78
Follow up time(m)
Number at risk
Wildtype 21 17 13 6 2 2 1 1 1 1 1 1 1 0
Alteration 12 10 4 3 1 0 0 0 0 0 0 0 0 0
0 6 12 18 24 30 36 42 48 54 60 66 72 78
Follow up time(m)

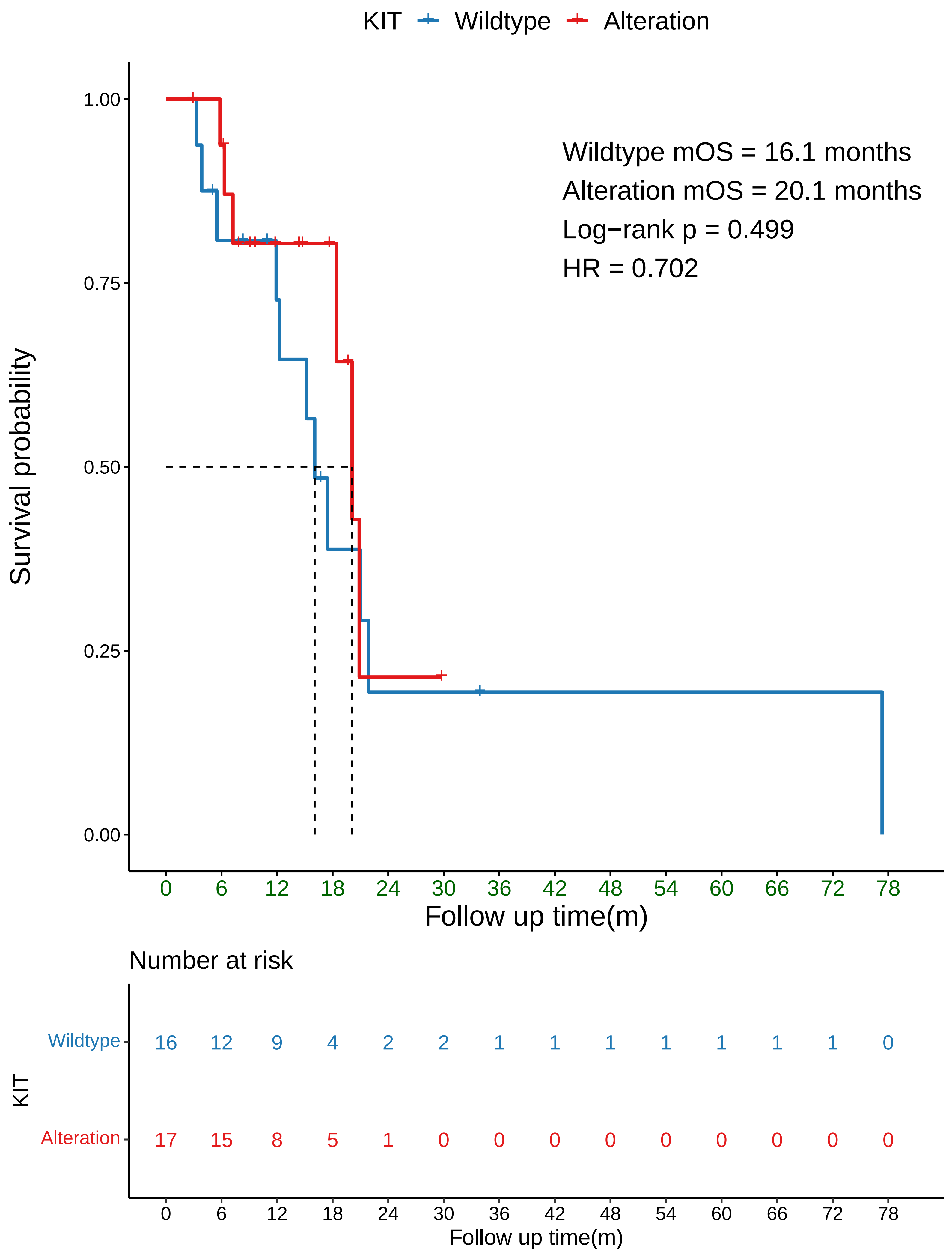

KIT Wildtype Alteration
Wildtype mOS = 16.1 months
Alteration mOS = 20.1 months
Log−rank p = 0.499
HR = 0.702
Survival probability
1.00
0.75
0.50
0.25
0.00
0 6 12 18 24 30 36 42 48 54 60 66 72 78
Follow up time(m)
Number at risk
KIT
Wildtype 16 12 9 4 2 2 1 1 1 1 1 1 1 0
Alteration 17 15 8 5 1 0 0 0 0 0 0 0 0 0
0 6 12 18 24 30 36 42 48 54 60 66 72 78
Follow up time(m)

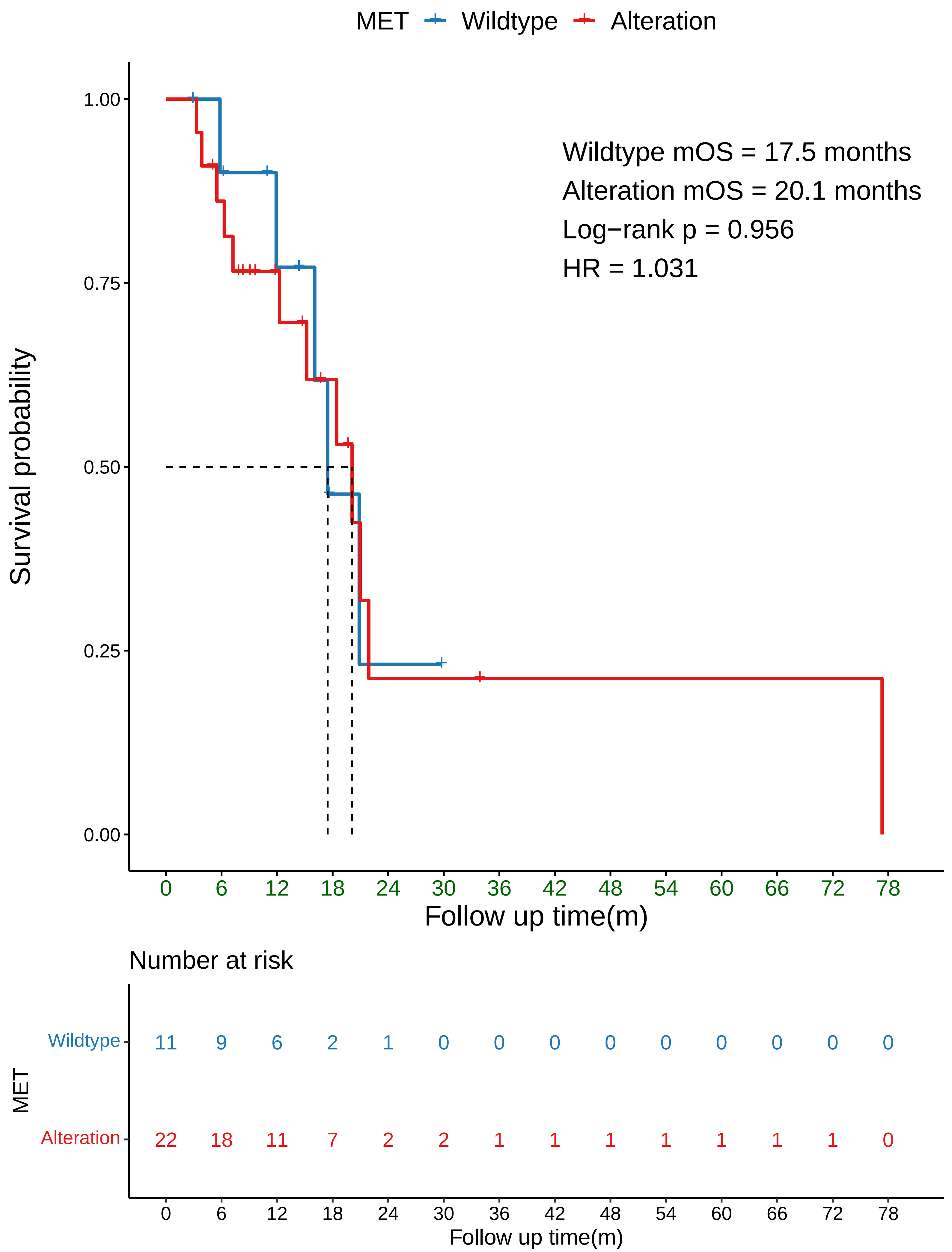

MET Wildtype Alteration
Wildtype mOS = 17.5 months
Alteration mOS = 20.1 months
Log−rank p = 0.956
HR = 1.031
Survival probability
1.00
0.75
0.50
0.25
0.00
0 6 12 18 24 30 36 42 48 54 60 66 72 78
Follow up time(m)
Number at risk
MET
Wildtype 11 9 6 2 1 0 0 0 0 0 0 0 0 0
Alteration 22 18 11 7 2 2 1 1 1 1 1 1 1 0
0 6 12 18 24 30 36 42 48 54 60 66 72 78
Follow up time(m)

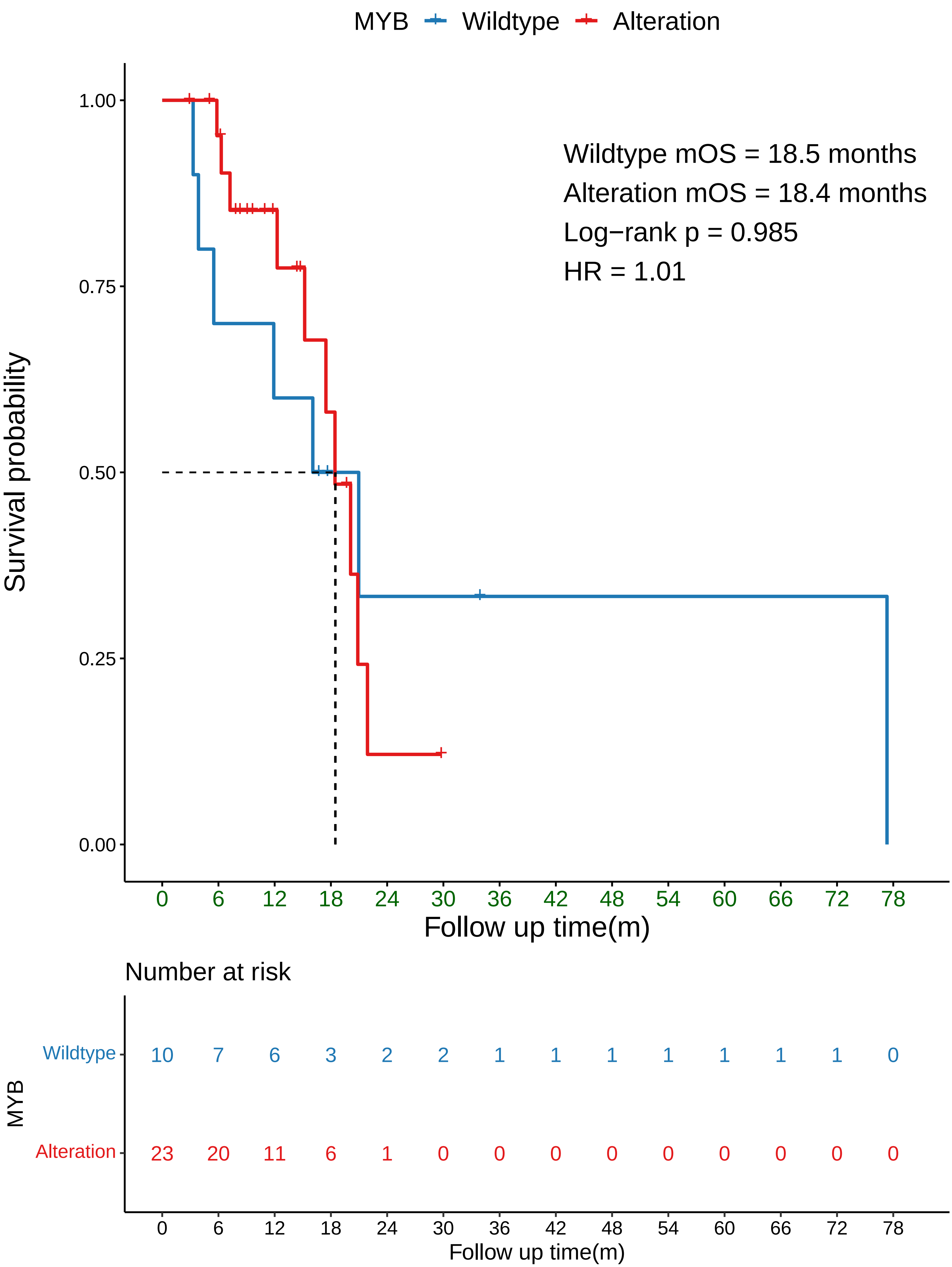

MYB
Wildtype
Alteration
Wildtype mOS = 18.5 months
Alteration mOS = 18.4 months
Log−rank p = 0.985
HR = 1.01
Survival probability
1.00
0.75
0.50
0.25
0.00
0 6 12 18 24 30 36 42 48 54 60 66 72 78
Follow up time(m)
Number at risk
MYB
Wildtype 10 7 6 3 2 2 1 1 1 1 1 1 1 0
Alteration 23 20 11 6 1 0 0 0 0 0 0 0 0 0
0 6 12 18 24 30 36 42 48 54 60 66 72 78
Follow up time(m)

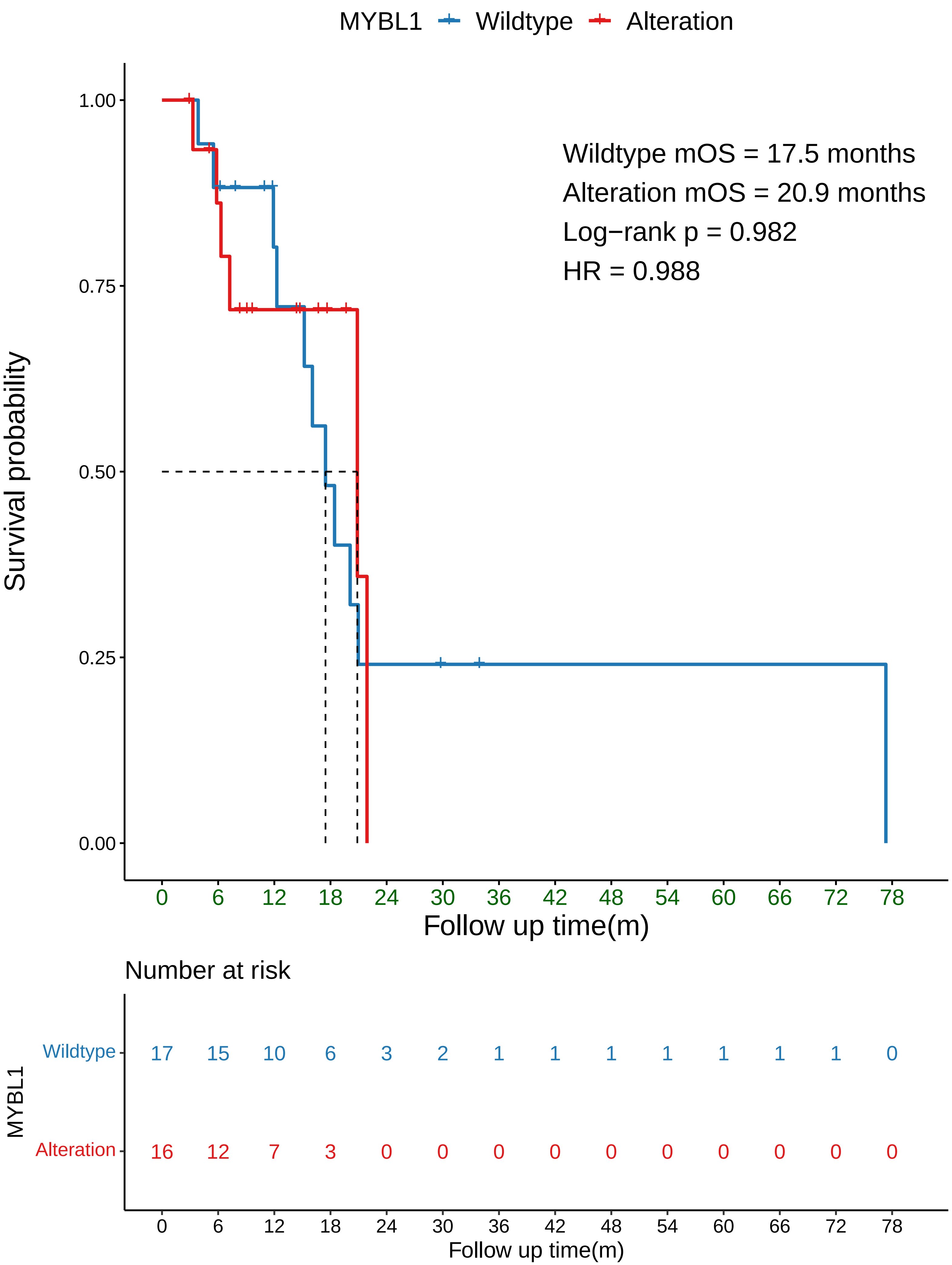

MYBL1
Wildtype
Alteration
Wildtype mOS = 17.5 months
Alteration mOS = 20.9 months
Log−rank p = 0.982
HR = 0.988
Survival probability
1.00
0.75
0.50
0.25
0.00
0 6 12 18 24 30 36 42 48 54 60 66 72 78
Follow up time(m)
Number at risk
Wildtype 17 15 10 6 3 2 1 1 1 1 1 1 1 0
Alteration 16 12 7 3 0 0 0 0 0 0 0 0 0 0
MYBL1
0 6 12 18 24 30 36 42 48 54 60 66 72 78
Follow up time(m)

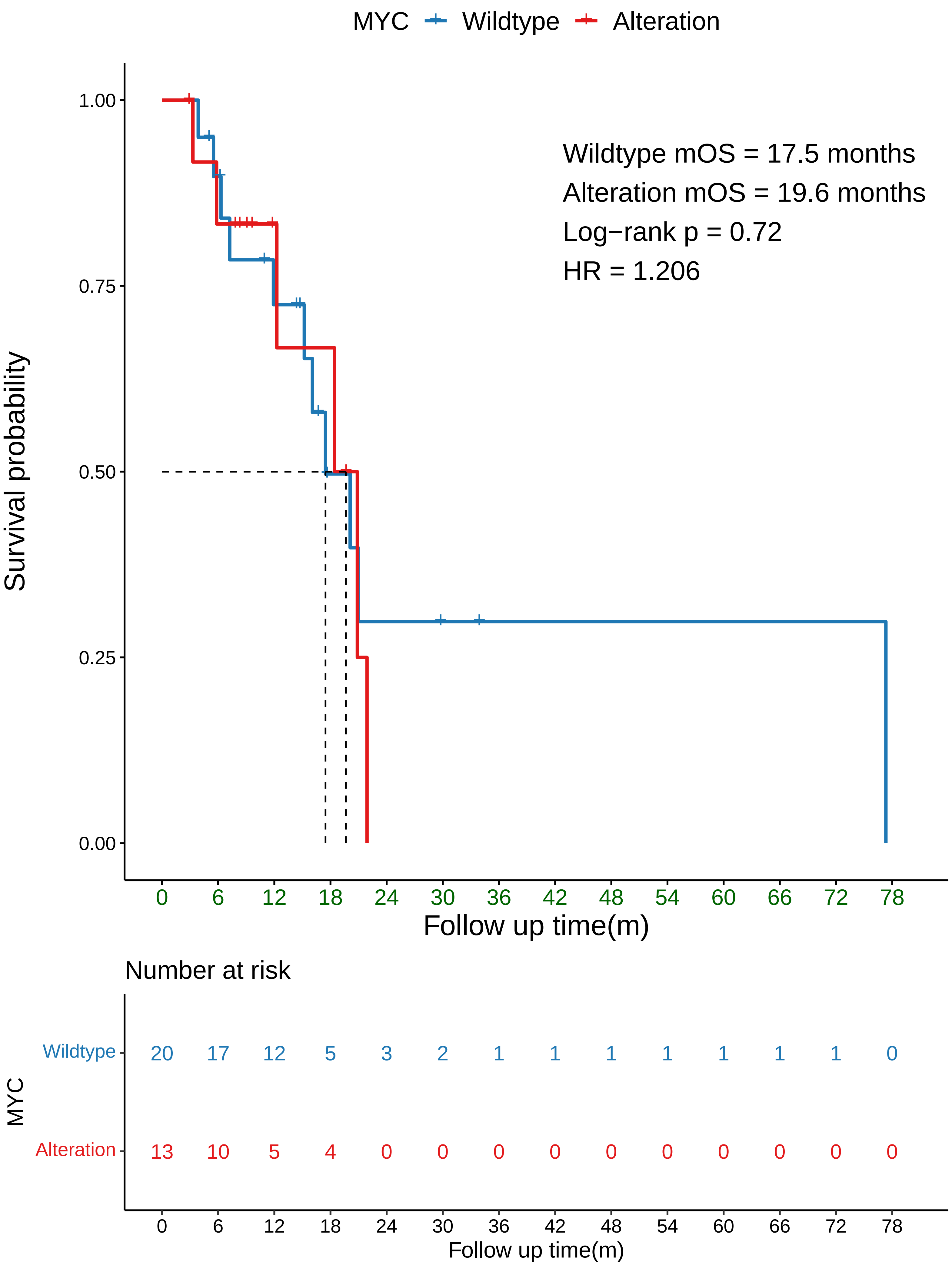

MYC Wildtype Alteration
Wildtype mOS = 17.5 months
Alteration mOS = 19.6 months
Log−rank p = 0.72
HR = 1.206
Survival probability
1.00
0.75
0.50
0.25
0.00
0 6 12 18 24 30 36 42 48 54 60 66 72 78
Follow up time(m)
Number at risk
MYC
Wildtype 20 17 12 5 3 2 1 1 1 1 1 1 1 0
Alteration 13 10 5 4 0 0 0 0 0 0 0 0 0 0
0 6 12 18 24 30 36 42 48 54 60 66 72 78
Follow up time(m)

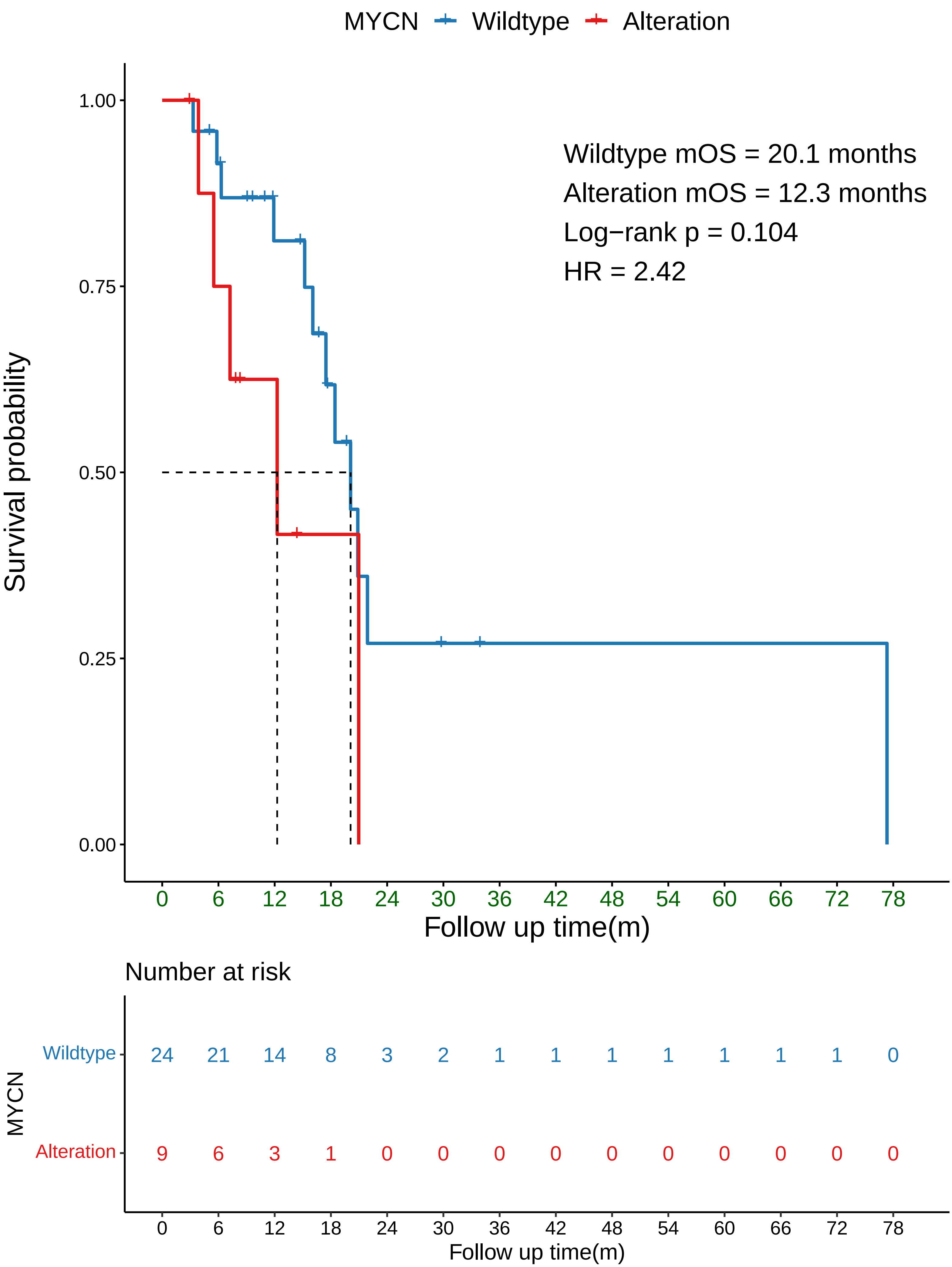
MYCN
Wildtype
Alteration
Wildtype mOS = 20.1 months
Alteration mOS = 12.3 months
Log−rank p = 0.104
HR = 2.42
Survival probability
1.00
0.75
0.50
0.25
0.00
0
6
12
18
24
30
36
42
48
54
60
66
72
78
Follow up time(m)
Number at risk
MYCN
Wildtype
24 21 14 8 3 2 1 1 1 1 1 1 1 0
Alteration
9 6 3 1 0 0 0 0 0 0 0 0 0 0
0
6
12
18
24
30
36
42
48
54
60
66
72
78
Follow up time(m)

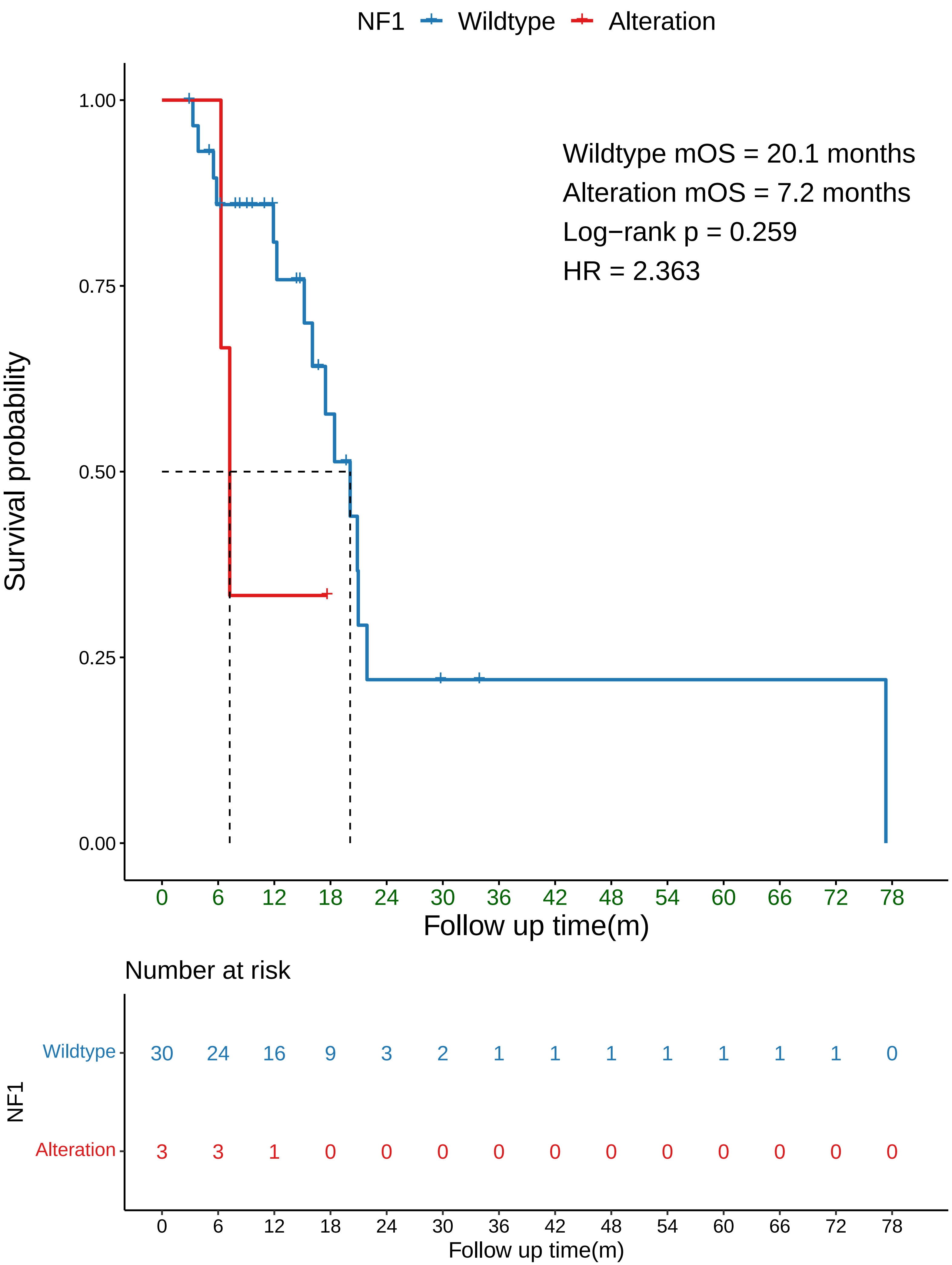

NF1 Wildtype Alteration
Wildtype mOS = 20.1 months
Alteration mOS = 7.2 months
Log−rank p = 0.259
HR = 2.363
Survival probability
1.00
0.75
0.50
0.25
0.00
0 6 12 18 24 30 36 42 48 54 60 66 72 78
Follow up time(m)
Number at risk
NF1
Wildtype 30 24 16 9 3 2 1 1 1 1 1 1 1 0
Alteration 3 3 1 0 0 0 0 0 0 0 0 0 0 0
0 6 12 18 24 30 36 42 48 54 60 66 72 78
Follow up time(m)

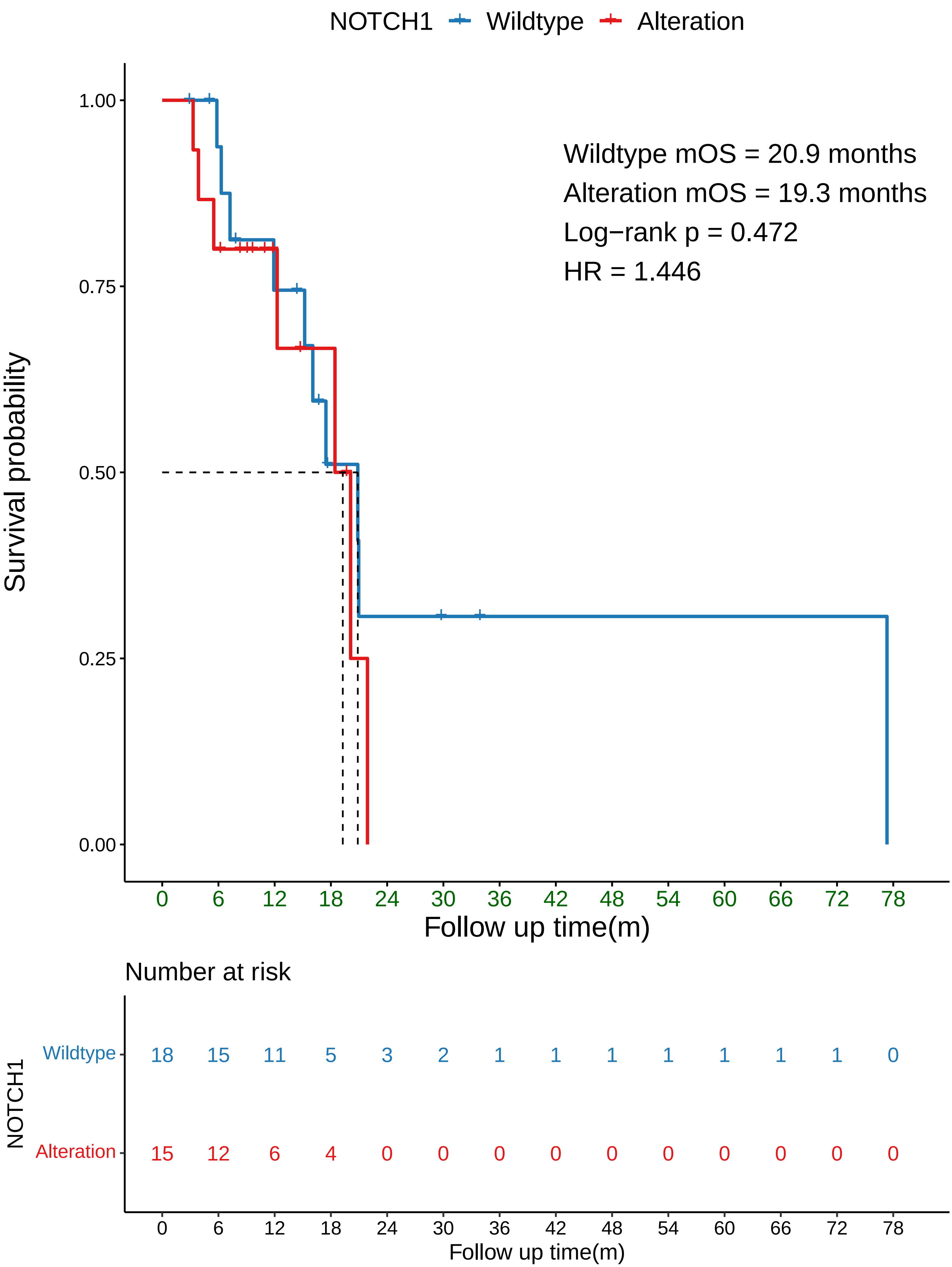
NOTCH1
Wildtype
Alteration
Wildtype mOS = 20.9 months
Alteration mOS = 19.3 months
Log−rank p = 0.472
HR = 1.446
Survival probability
1.00
0.75
0.50
0.25
0.00
0 6 12 18 24 30 36 42 48 54 60 66 72 78
Follow up time(m)
Number at risk
NOTCH1
Wildtype 18 15 11 5 3 2 1 1 1 1 1 1 1 0
Alteration 15 12 6 4 0 0 0 0 0 0 0 0 0 0
0 6 12 18 24 30 36 42 48 54 60 66 72 78
Follow up time(m)

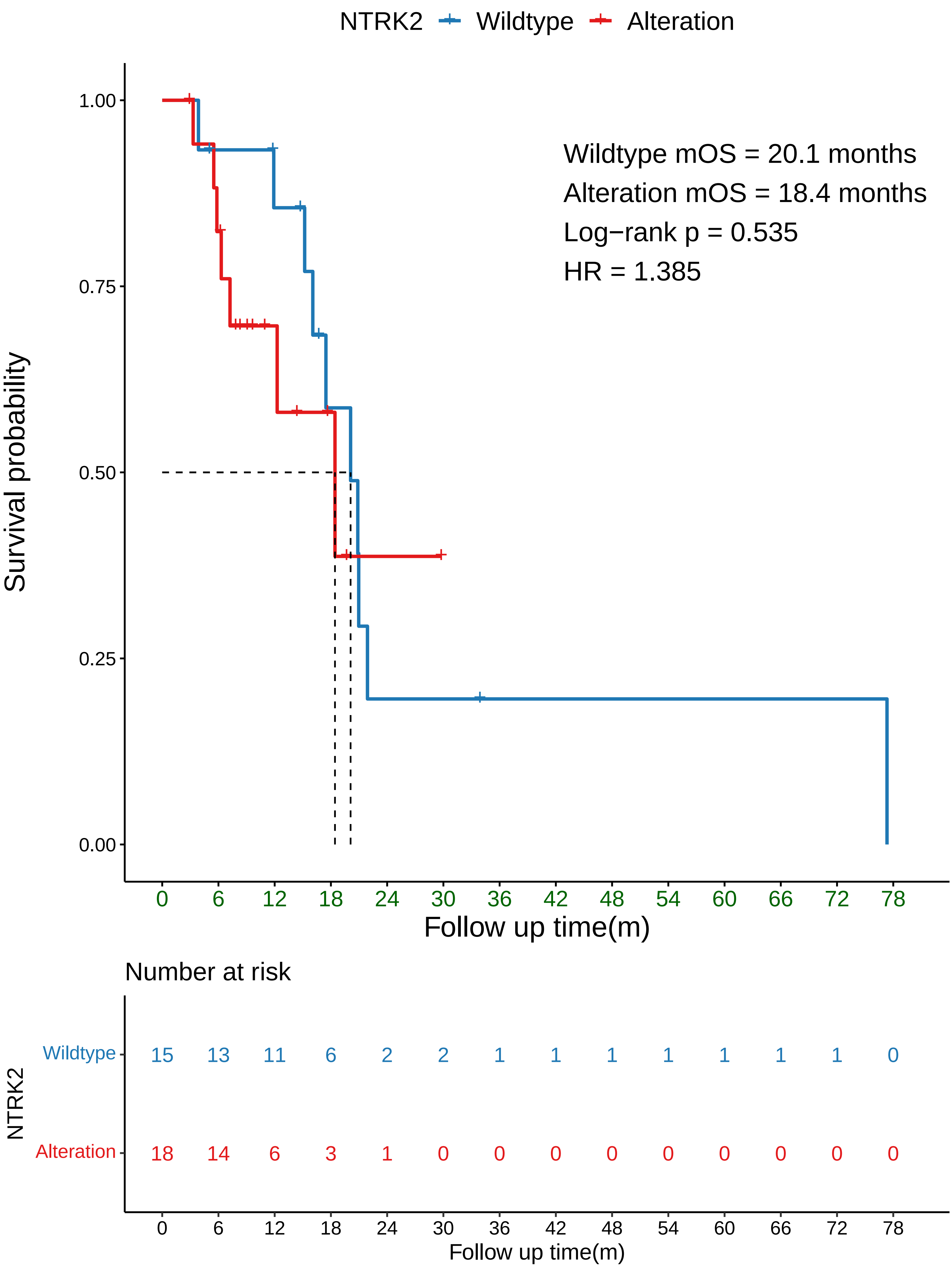
NTRK2 Wildtype Alteration
Wildtype mOS = 20.1 months
Alteration mOS = 18.4 months
Log−rank p = 0.535
HR = 1.385
Survival probability
1.00
0.75
0.50
0.25
0.00
0 6 12 18 24 30 36 42 48 54 60 66 72 78
Follow up time(m)
Number at risk
NTRK2
Wildtype 15 13 11 6 2 2 1 1 1 1 1 1 1 0
Alteration 18 14 6 3 1 0 0 0 0 0 0 0 0 0
0 6 12 18 24 30 36 42 48 54 60 66 72 78
Follow up time(m)

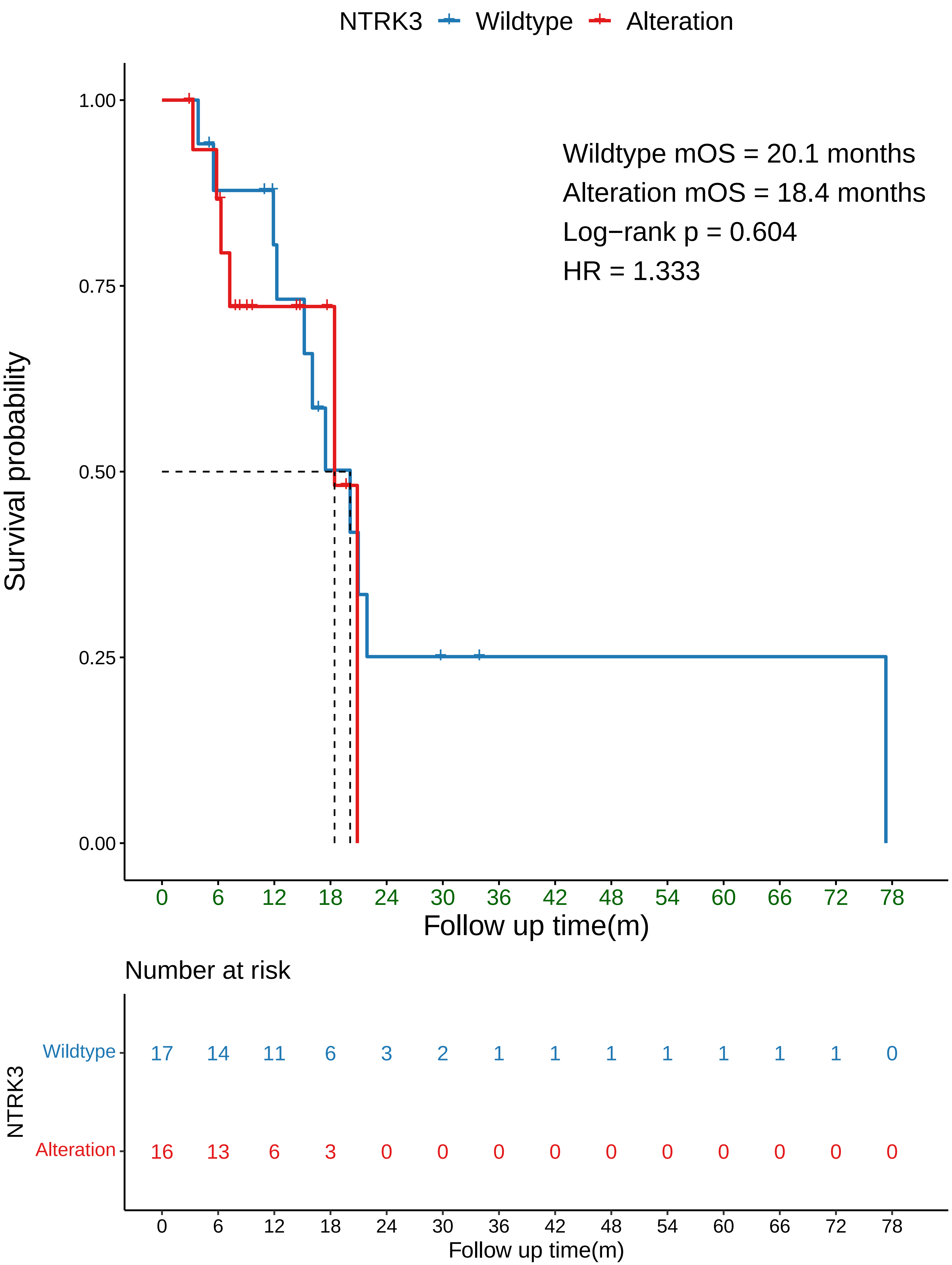
NTRK3 Wildtype Alteration
Wildtype mOS = 20.1 months
Alteration mOS = 18.4 months
Log−rank p = 0.604
HR = 1.333
Survival probability
1.00
0.75
0.50
0.25
0.00
0 6 12 18 24 30 36 42 48 54 60 66 72 78
Follow up time(m)
Number at risk
NTRK3
Wildtype 17 14 11 6 3 2 1 1 1 1 1 1 1 0
Alteration 16 13 6 3 0 0 0 0 0 0 0 0 0 0
0 6 12 18 24 30 36 42 48 54 60 66 72 78
Follow up time(m)

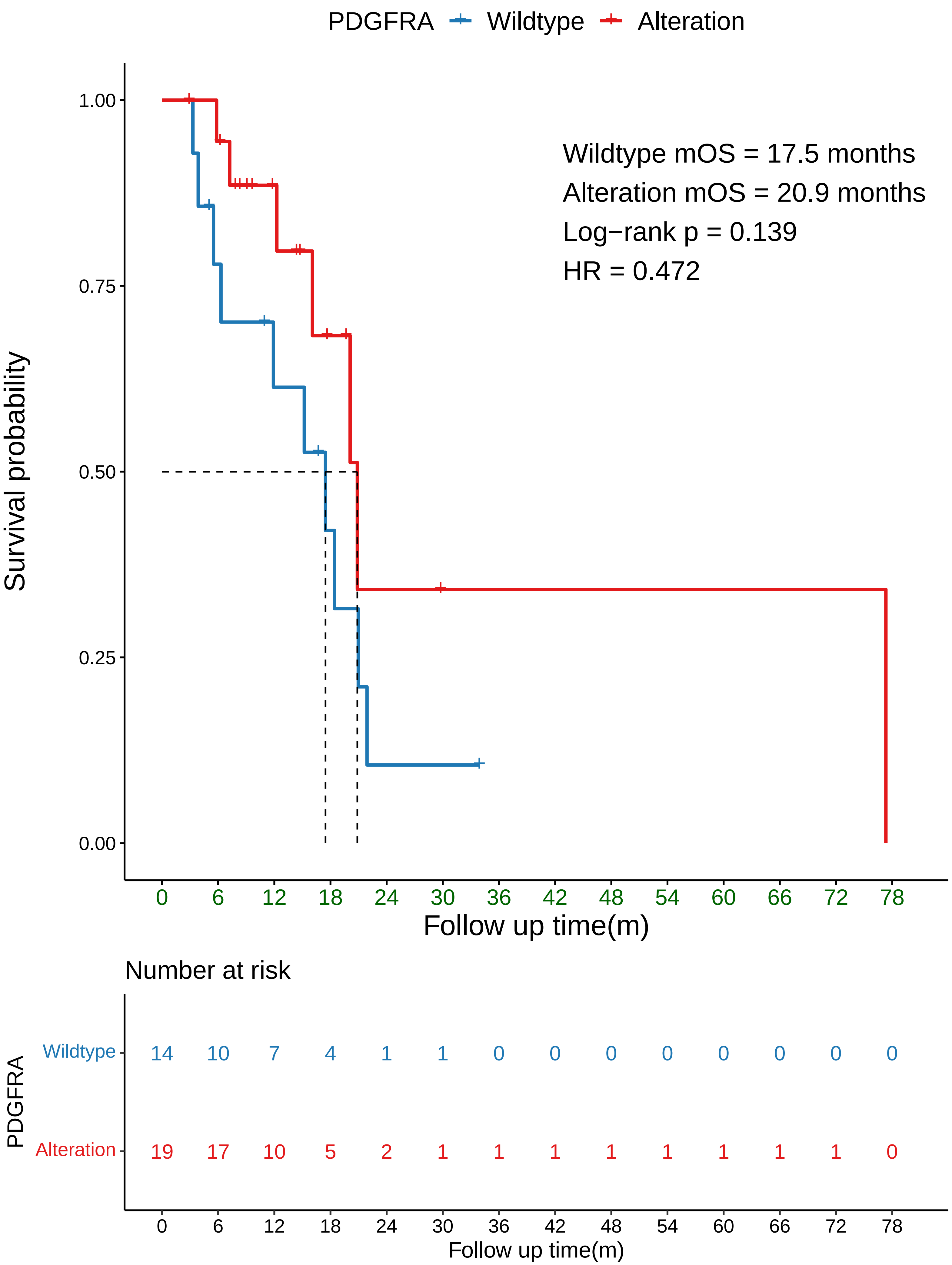

PDGFRA
Wildtype
Alteration
Wildtype mOS = 17.5 months
Alteration mOS = 20.9 months
Log−rank p = 0.139
HR = 0.472
Survival probability
1.00
0.75
0.50
0.25
0.00
0 6 12 18 24 30 36 42 48 54 60 66 72 78
Follow up time(m)
Number at risk
PDGFRA
Wildtype 14 10 7 4 1 1 0 0 0 0 0 0 0 0
Alteration 19 17 10 5 2 1 1 1 1 1 1 1 1 0
0 6 12 18 24 30 36 42 48 54 60 66 72 78
Follow up time(m)

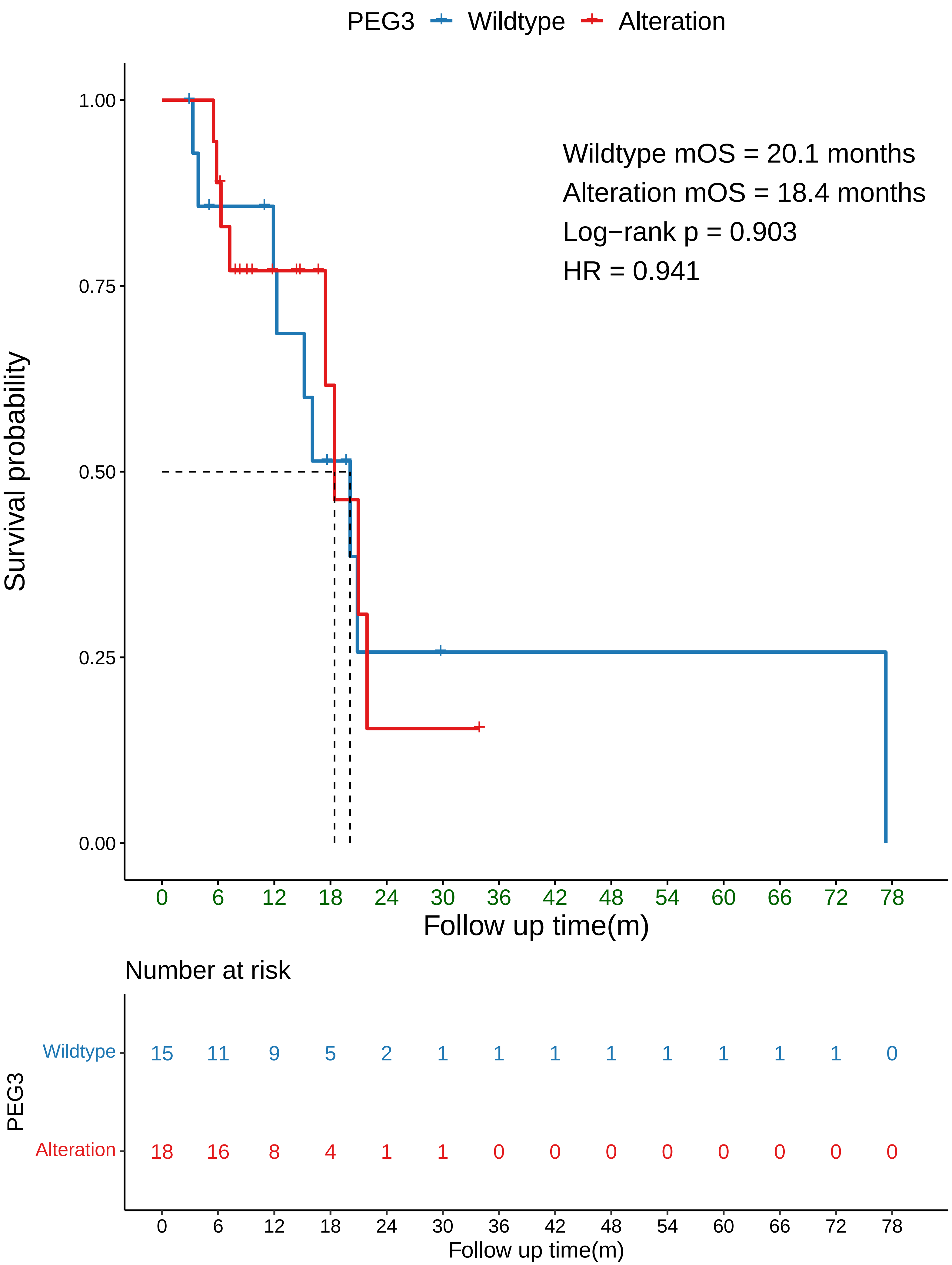

PEG3
Wildtype
Alteration
Wildtype mOS = 20.1 months
Alteration mOS = 18.4 months
Log−rank p = 0.903
HR = 0.941
Survival probability
1.00
0.75
0.50
0.25
0.00
0
6
12
18
24
30
36
42
48
54
60
66
72
78
Follow up time(m)
Number at risk
Wildtype
15 11 9 5 2 1 1 1 1 1 1 1 1 0
Alteration
18 16 8 4 1 1 0 0 0 0 0 0 0 0
PEG3
Follow up time(m)

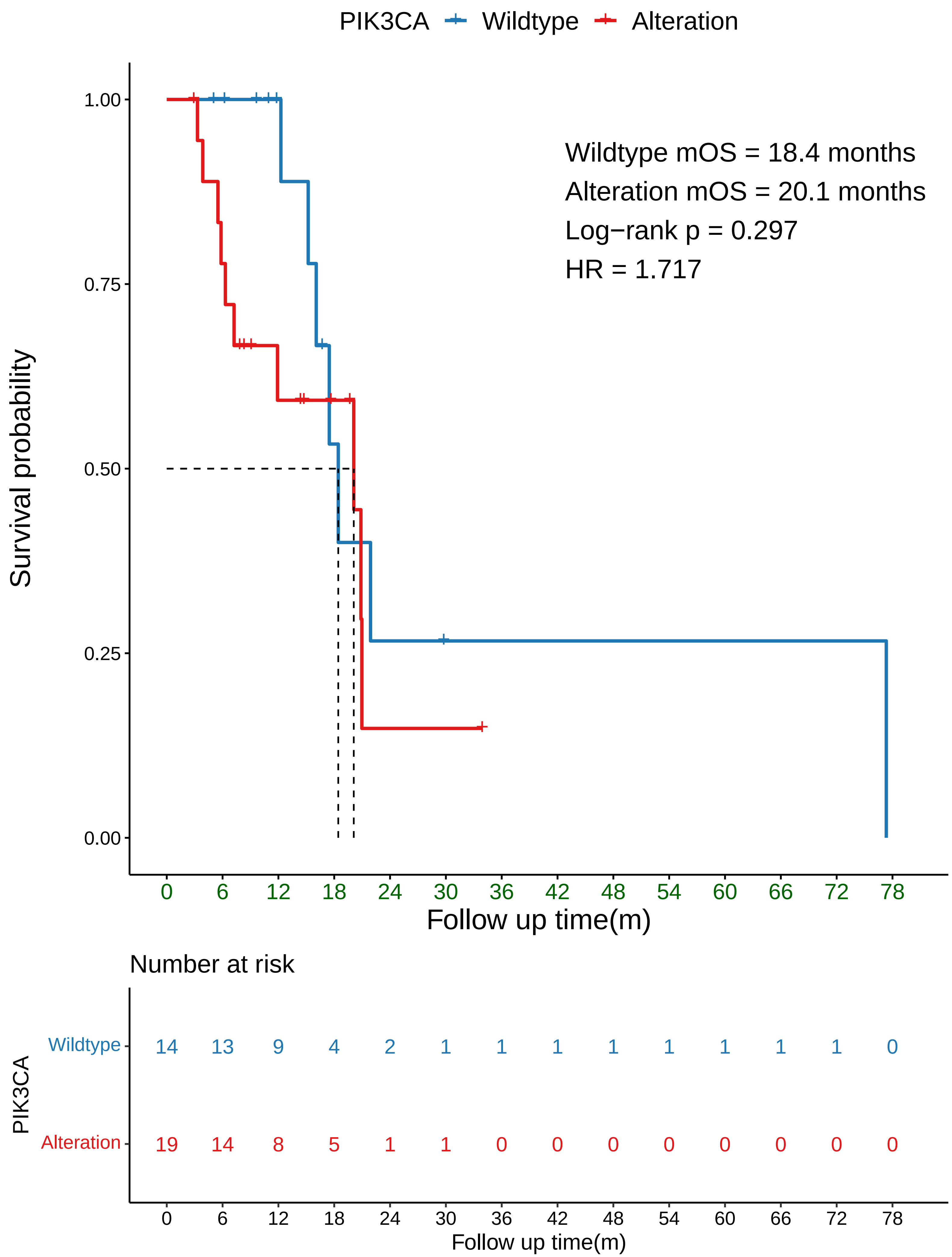
PIK3CA
Wildtype
Alteration
Wildtype mOS = 18.4 months
Alteration mOS = 20.1 months
Log−rank p = 0.297
HR = 1.717
Survival probability
1.00
0.75
0.50
0.25
0.00
0
6
12
18
24
30
36
42
48
54
60
66
72
78
Follow up time(m)
Number at risk
PIK3CA
Wildtype
14 13 9 4 2 1 1 1 1 1 1 1 1 0
Alteration
19 14 8 5 1 1 0 0 0 0 0 0 0 0
Follow up time(m)

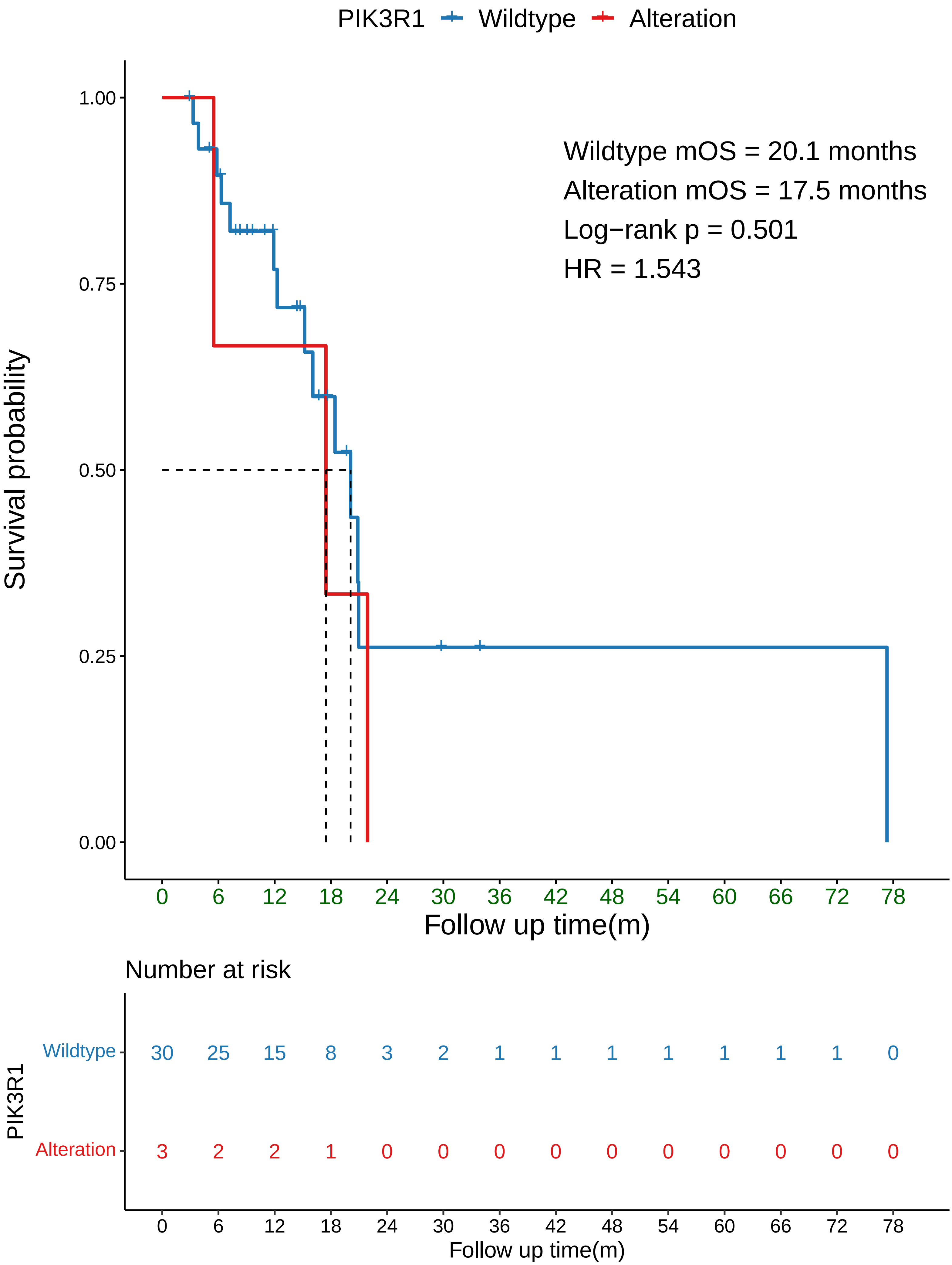

PIK3R1
Wildtype
Alteration
Wildtype mOS = 20.1 months
Alteration mOS = 17.5 months
Log−rank p = 0.501
HR = 1.543
Survival probability
1.00
0.75
0.50
0.25
0.00
0 6 12 18 24 30 36 42 48 54 60 66 72 78
Follow up time(m)
Number at risk
PIK3R1
Wildtype 30 25 15 8 3 2 1 1 1 1 1 1 1 0
Alteration 3 2 2 1 0 0 0 0 0 0 0 0 0 0
0 6 12 18 24 30 36 42 48 54 60 66 72 78
Follow up time(m)

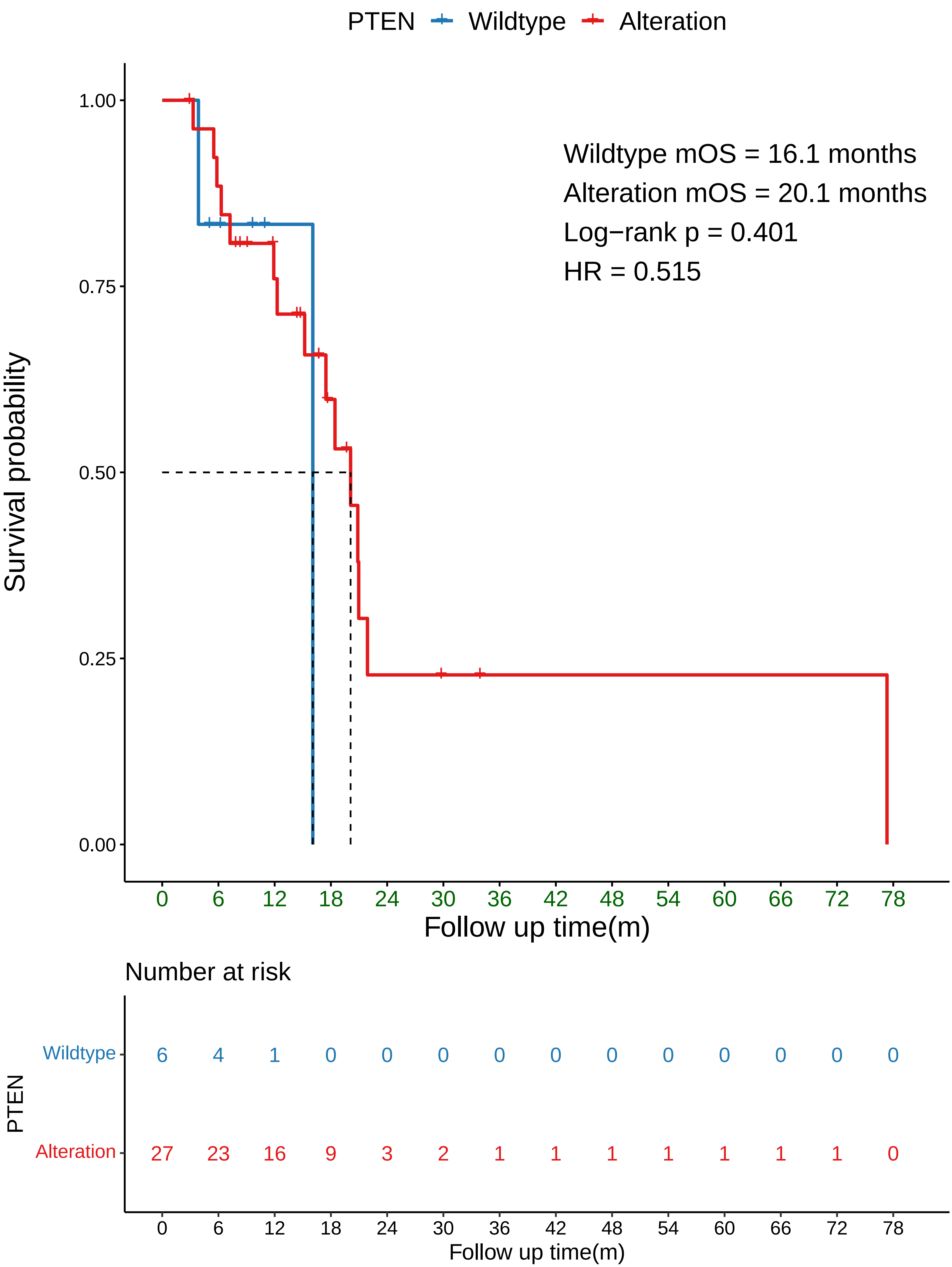
PTEN Wildtype Alteration
Wildtype mOS = 16.1 months
Alteration mOS = 20.1 months
Log−rank p = 0.401
HR = 0.515
Survival probability
1.00
0.75
0.50
0.25
0.00
0 6 12 18 24 30 36 42 48 54 60 66 72 78
Follow up time(m)
Number at risk
PTEN
Wildtype 6 4 1 0 0 0 0 0 0 0 0 0 0 0
Alteration 27 23 16 9 3 2 1 1 1 1 1 1 1 0
0 6 12 18 24 30 36 42 48 54 60 66 72 78
Follow up time(m)

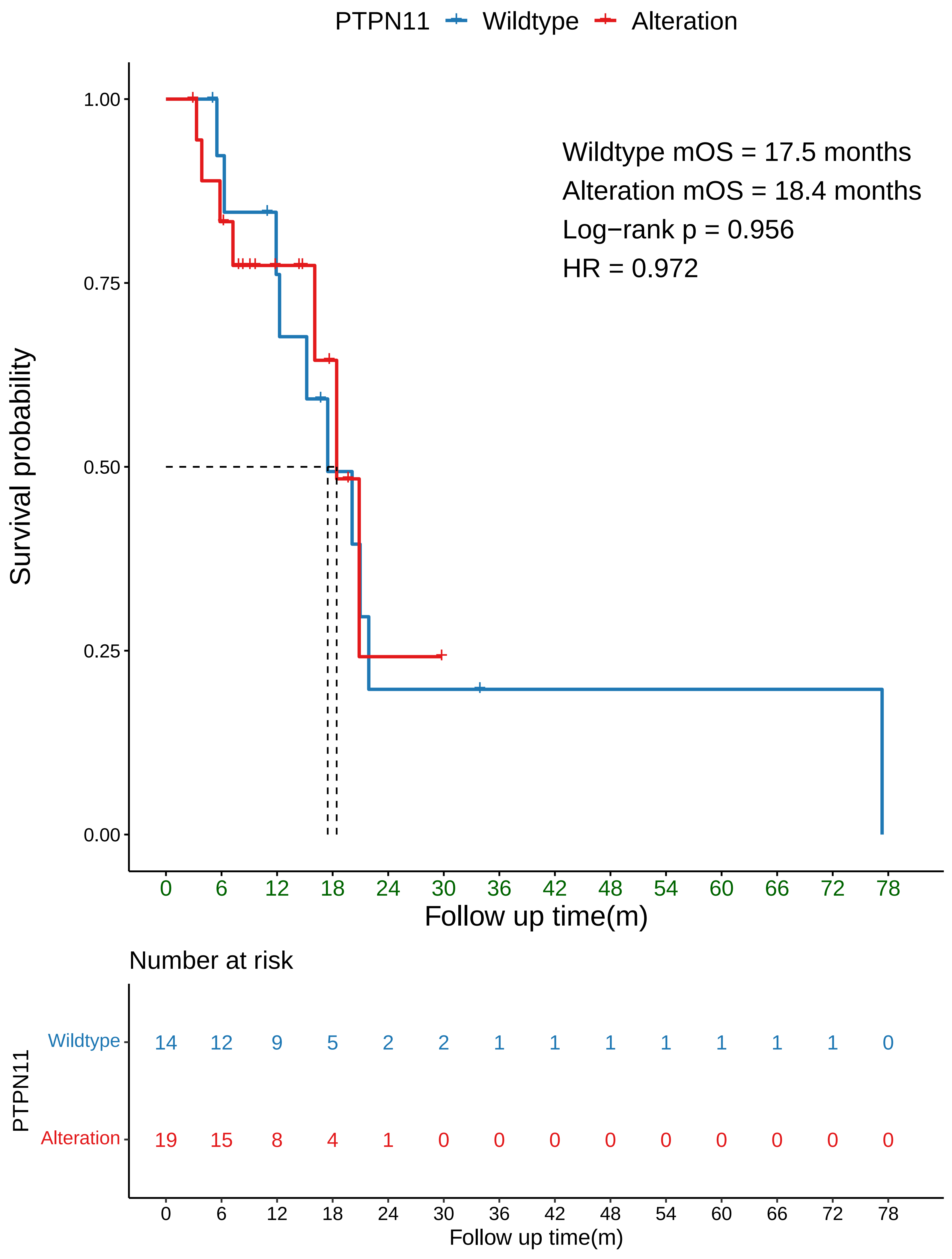
PTPN11
Wildtype
Alteration
Wildtype mOS = 17.5 months
Alteration mOS = 18.4 months
Log−rank p = 0.956
HR = 0.972
Survival probability
1.00
0.75
0.50
0.25
0.00
0 6 12 18 24 30 36 42 48 54 60 66 72 78
Follow up time(m)
Number at risk
PTPN11
Wildtype 14 12 9 5 2 2 1 1 1 1 1 1 1 0
Alteration 19 15 8 4 1 0 0 0 0 0 0 0 0 0
0 6 12 18 24 30 36 42 48 54 60 66 72 78
Follow up time(m)

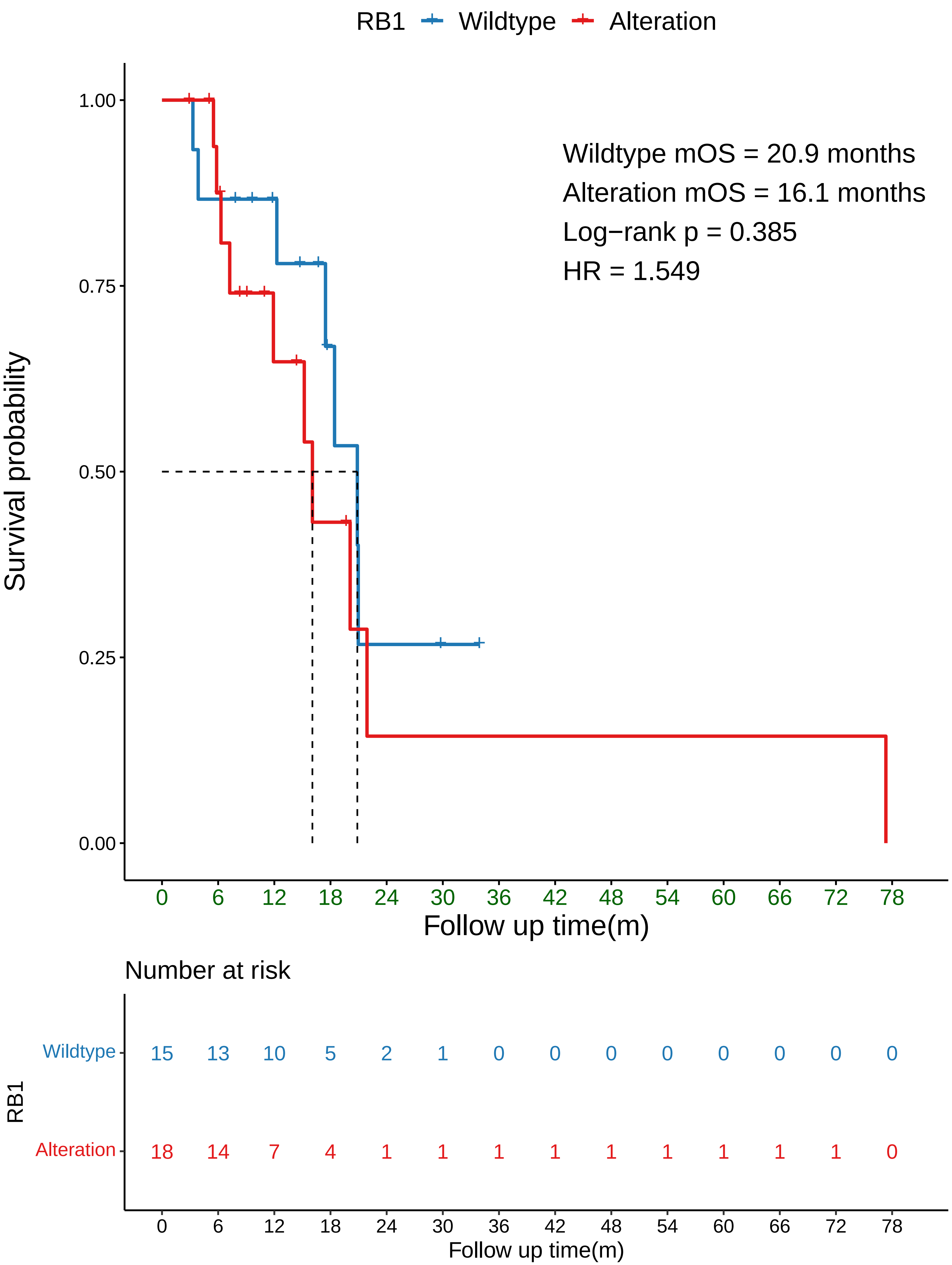
RB1 Wildtype Alteration
Wildtype mOS = 20.9 months
Alteration mOS = 16.1 months
Log−rank p = 0.385
HR = 1.549
Survival probability
1.00
0.75
0.50
0.25
0.00
0 6 12 18 24 30 36 42 48 54 60 66 72 78
Follow up time(m)
Number at risk
RB1
Wildtype 15 13 10 5 2 1 0 0 0 0 0 0 0 0
Alteration 18 14 7 4 1 1 1 1 1 1 1 1 1 0
0 6 12 18 24 30 36 42 48 54 60 66 72 78
Follow up time(m)

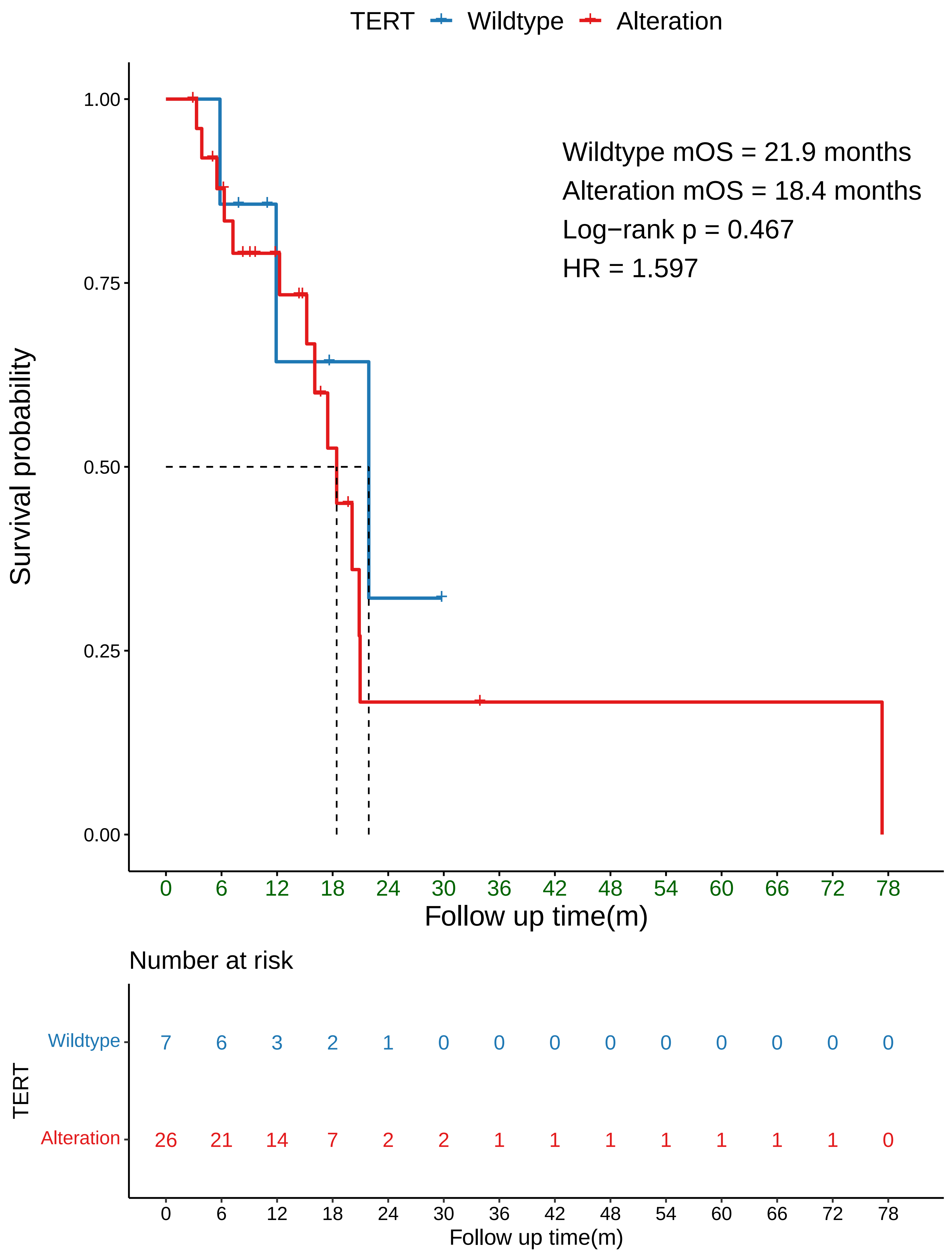
TERT Wildtype Alteration
Wildtype mOS = 21.9 months
Alteration mOS = 18.4 months
Log−rank p = 0.467
HR = 1.597
Survival probability
1.00
0.75
0.50
0.25
0.00
0 6 12 18 24 30 36 42 48 54 60 66 72 78
Follow up time(m)
Number at risk
TERT
Wildtype 7 6 3 2 1 0 0 0 0 0 0 0 0 0
Alteration 26 21 14 7 2 2 1 1 1 1 1 1 1 0
0 6 12 18 24 30 36 42 48 54 60 66 72 78
Follow up time(m)

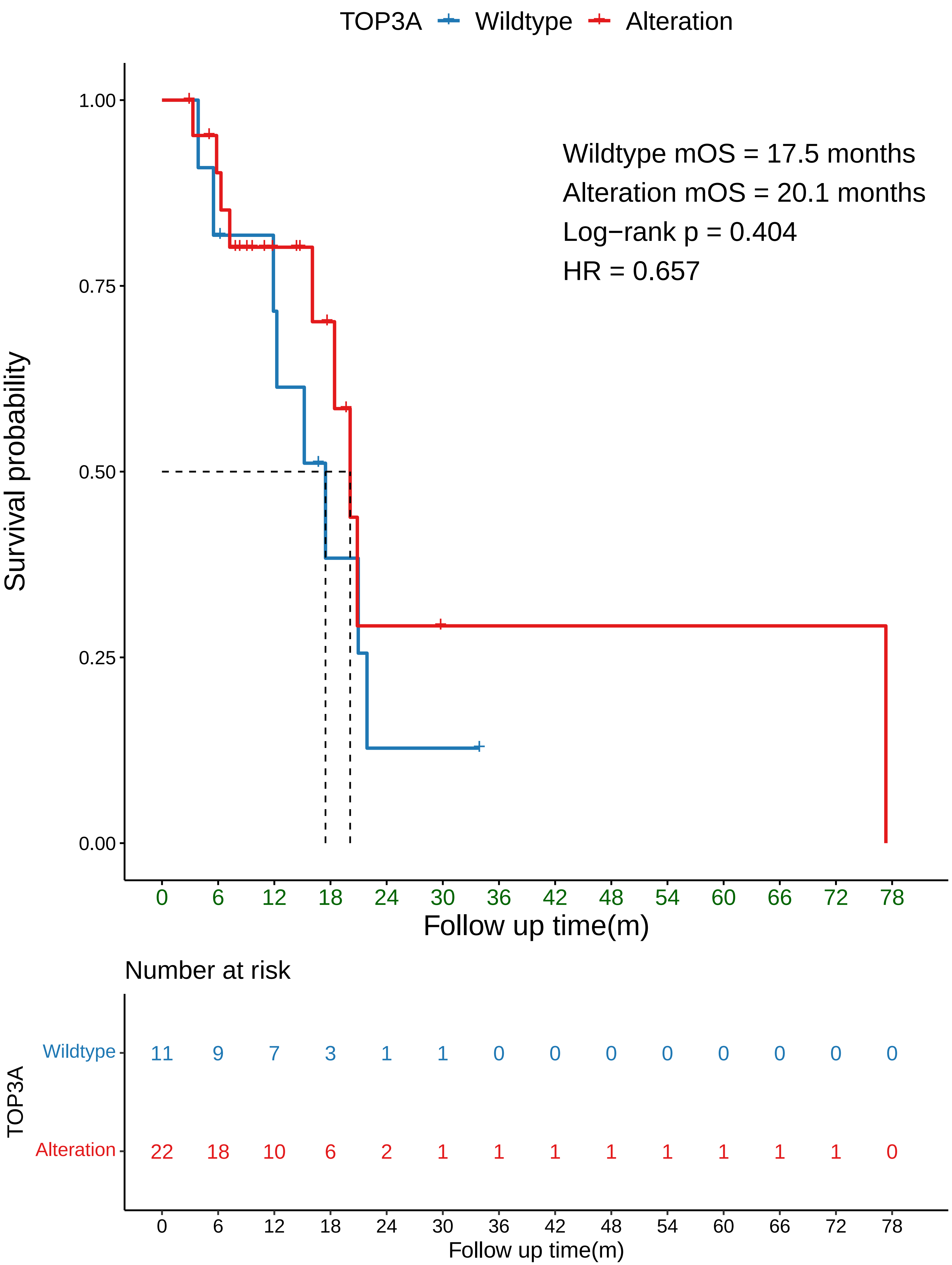

TOP3A
Wildtype
Alteration
Wildtype mOS = 17.5 months
Alteration mOS = 20.1 months
Log−rank p = 0.404
HR = 0.657
Survival probability
1.00
0.75
0.50
0.25
0.00
0
6
12
18
24
30
36
42
48
54
60
66
72
78
Follow up time(m)
Number at risk
TOP3A
Wildtype 11 9 7 3 1 1 0 0 0 0 0 0 0 0
Alteration 22 18 10 6 2 1 1 1 1 1 1 1 1 0
Follow up time(m)

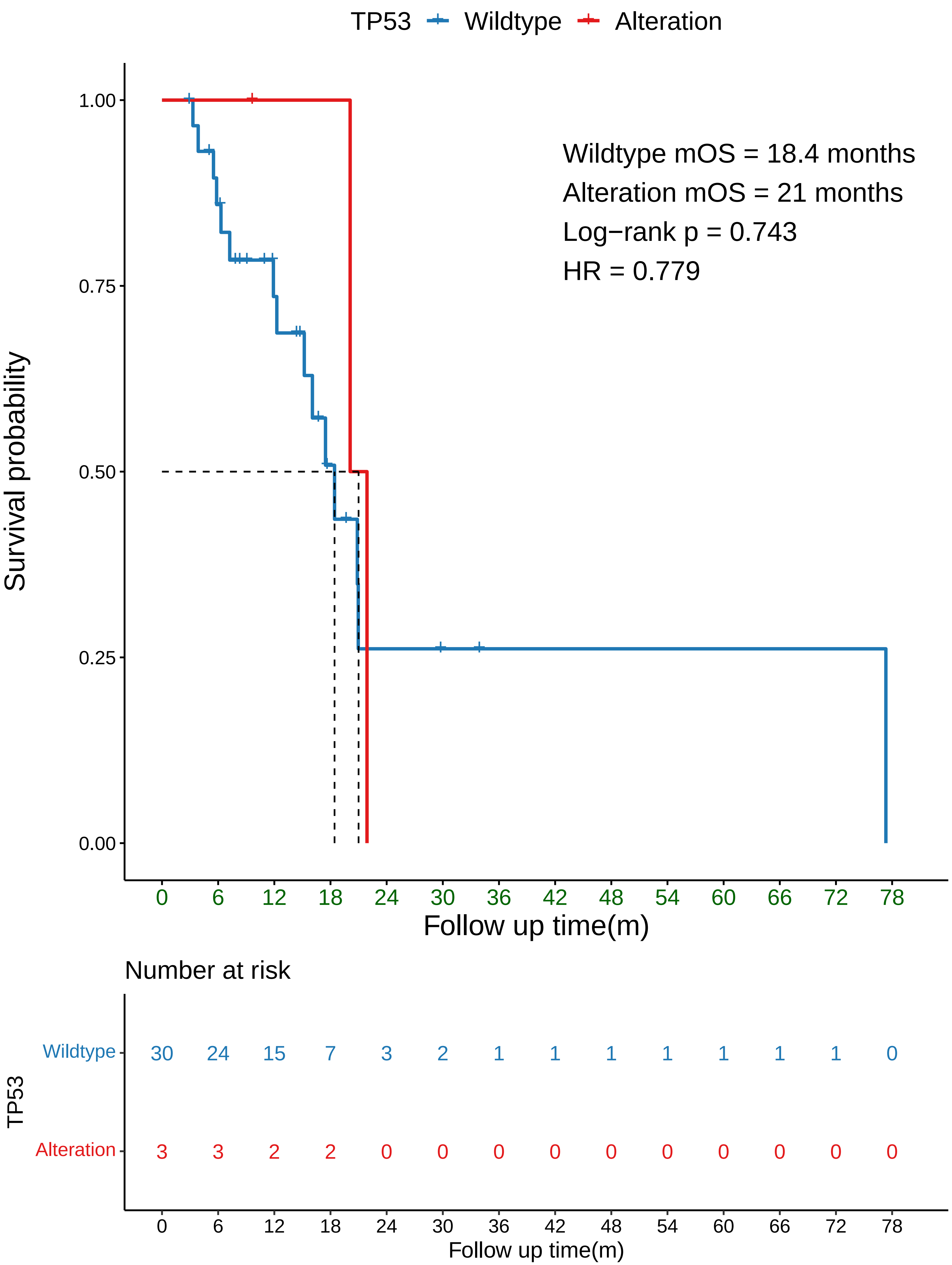

TP53
Wildtype
Alteration
Wildtype mOS = 18.4 months
Alteration mOS = 21 months
Log−rank p = 0.743
HR = 0.779
Survival probability
1.00
0.75
0.50
0.25
0.00
0 6 12 18 24 30 36 42 48 54 60 66 72 78
Follow up time(m)
Number at risk
TP53
Wildtype 30 24 15 7 3 2 1 1 1 1 1 1 1 0
Alteration 3 3 2 2 0 0 0 0 0 0 0 0 0 0
0 6 12 18 24 30 36 42 48 54 60 66 72 78
Follow up time(m)
